# Fanzor is a eukaryotic programmable RNA-guided endonuclease

**DOI:** 10.1038/s41586-023-06356-2

**Published:** 2023-06-28

**Authors:** Makoto Saito, Peiyu Xu, Guilhem Faure, Samantha Maguire, Soumya Kannan, Han Altae-Tran, Sam Vo, AnAn Desimone, Rhiannon K. Macrae, Feng Zhang

**Affiliations:** 1 Broad Institute of MIT and Harvard, Cambridge, MA USA; 2 McGovern Institute for Brain Research at MIT, Cambridge, MA USA; 3 Department of Brain and Cognitive Science, Massachusetts Institute of Technology, Cambridge, MA USA; 4 Department of Biological Engineering, Massachusetts Institute of Technology, Cambridge, MA USA; 5 Howard Hughes Medical Institute, Cambridge, MA USA

**Keywords:** Biochemistry, Structural biology

## Abstract

RNA-guided systems, which use complementarity between a guide RNA and target nucleic acid sequences for recognition of genetic elements, have a central role in biological processes in both prokaryotes and eukaryotes. For example, the prokaryotic CRISPR–Cas systems provide adaptive immunity for bacteria and archaea against foreign genetic elements. Cas effectors such as Cas9 and Cas12 perform guide-RNA-dependent DNA cleavage^1^. Although a few eukaryotic RNA-guided systems have been studied, including RNA interference^2^ and ribosomal RNA modification^3^, it remains unclear whether eukaryotes have RNA-guided endonucleases. Recently, a new class of prokaryotic RNA-guided systems (termed OMEGA) was reported^4,5^. The OMEGA effector TnpB is the putative ancestor of Cas12 and has RNA-guided endonuclease activity^4,6^. TnpB may also be the ancestor of the eukaryotic transposon-encoded Fanzor (Fz) proteins^4,7^, raising the possibility that eukaryotes are also equipped with CRISPR–Cas or OMEGA-like programmable RNA-guided endonucleases. Here we report the biochemical characterization of Fz, showing that it is an RNA-guided DNA endonuclease. We also show that Fz can be reprogrammed for human genome engineering applications. Finally, we resolve the structure of *Spizellomyces punctatus* Fz at 2.7 Å using cryogenic electron microscopy, showing the conservation of core regions among Fz, TnpB and Cas12, despite diverse cognate RNA structures. Our results show that Fz is a eukaryotic OMEGA system, demonstrating that RNA-guided endonucleases are present in all three domains of life.

## Abstract (web summary)

Fanzor is shown to be an RNA-guided DNA endonuclease, demonstrating that such endonucleases are found in all domains of life and indicating a potential new tool for genome engineering applications.

## Main

Fanzor (Fz) was reported in 2013 to be a eukaryotic TnpB-IS200/IS605-like protein encoded by transposable elements, and it was initially suggested that Fz proteins (and prokaryotic TnpBs) regulate transposable element activity, possibly through methyltransferase activity^7^. More recently, TnpB was reported to be part of a new class of RNA-guided systems termed OMEGA (Obligate Mobile Element-guided Activity)^4,6^. OMEGA systems encompass an RNA-guided endonuclease protein (that is, TnpB, IscB or IsrB) and a non-coding RNA (ncRNA) transcribed from the transposon end region (called ωRNA)^4^. OMEGA systems are the ancestors of CRISPR–Cas systems, and TnpB evolved into the single RNA-guided endonuclease Cas12. TnpB also has remote homology with Fz^4^. These findings raise the possibility that Fz may be a eukaryotic type of CRISPR–Cas or OMEGA system. By combining phylogenomic, biochemical and structural studies, we sought to determine the enzymatic activity and mechanism of Fz and reprogramme it for human genome editing.

## Fz diversity

**Fig. 1:**
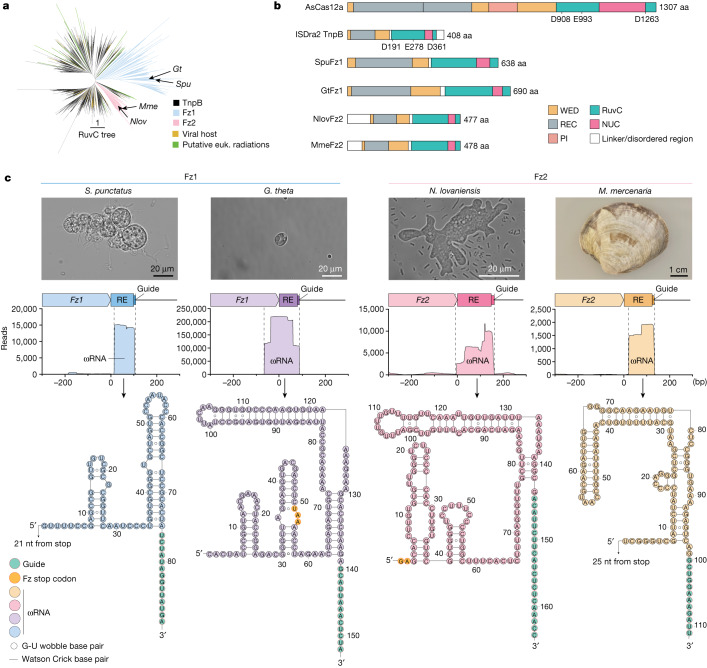
Phylogenetic analysis of Fz. **a**, Unrooted phylogenetic tree from representatives mined from Fz and TnpB. Arrows indicate *Fz* genes experimentally characterized in this study (*SpuFz1*, *GtFz1*, *NlovFz2* and *MmeFz2*). A detailed tree is shown in Extended Data Fig. 1. **b**, Domain architectures of AsCas12a, ISDra2 TnpB, SpuFz1, GtFz1, NlovFz2 and MmeFz2 determined from structural analysis (Extended Data Fig. 2). **c**, Top, micrographs of *S. punctatus*, *G. theta* and *N. lovaniensis* and a photograph of *M. mercenaria*. Representative images from three independent cultures are shown. Middle, small RNA-seq for RNPs of four representative *Fz* orthologues expressed in *S. cerevisiae* (*n* = 3 independent technical replicates). Bottom, secondary structure prediction of ωRNAs for the four representative orthologues. When the ωRNA overlaps the *Fz* gene, the stop codon is shown in orange; when there is no overlap, the distance to the stop codon is indicated with an arrow. The guide region is shown in green and oriented vertically for comparison. euk., eukaryotic; PI, PAM interacting; RE, right end.

**Extended Data Fig. 1:**
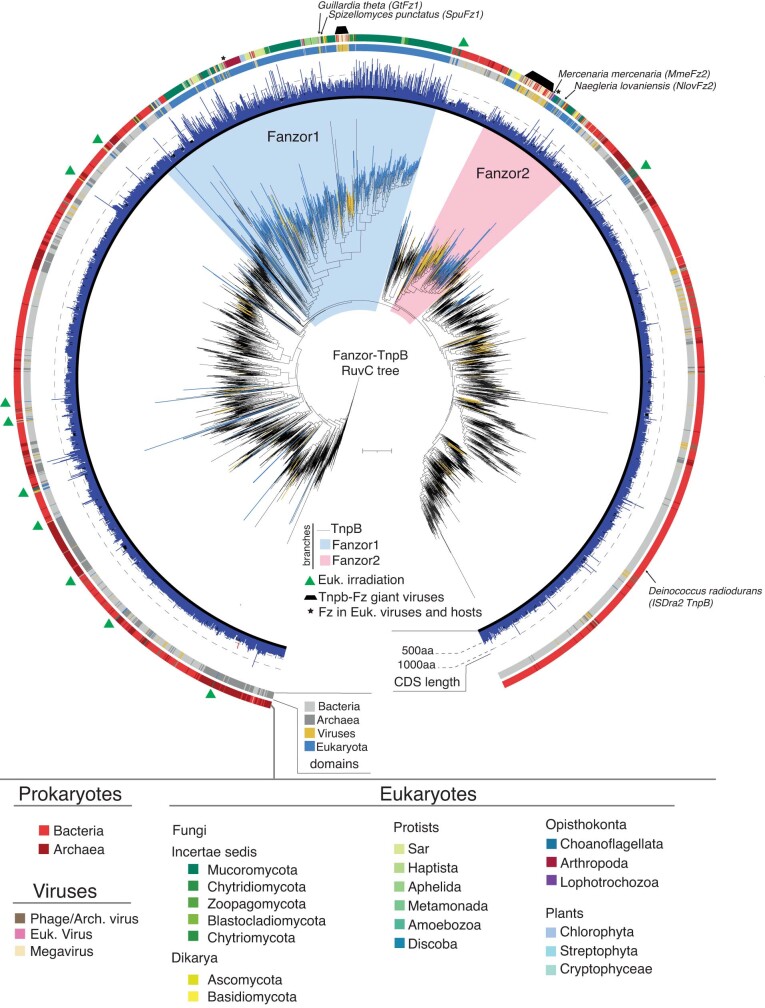
Phylogenetic tree of Fanzor and TnpB. Phylogenetic tree built from the RuvC region of hits detected from structural and profile mining of Fanzor. Blue, black and yellow leaves indicate the domain annotation of the contig where the hit is found respectively eukaryotes, viruses and prokaryotes. Fanzor1 and Fanzor2 clades are shown respectively in blue and pink. Fanzors and TnpB of interest are indicated by arrows. The bars forming the blue inner ring are proportional to the size of the Fanzors in aa as annotated in the database. The middle ring indicates the domains of life from which the Fanzor/TnpB is found (light gray: bacteria, dark gray: archaea, yellow: viruses, blue: eukaryotes). The outer ring displays the taxonomy of the organism in which the Fanzor/TnpB is found (red: bacteria, dark red: archaea, brown: phage and archaeal viruses, pink: eukaryotic viruses, beige: giant viruses, dark green to yellow gradient: fungi, light green gradient: protists, dark blue: opisthokonta (choanoflagellata), crimson: arthropoda, purple: mollusks, and light blue to dark blue gradient: plants with Chlorophyta, Streptophyta, and Cryptophyceae. The green triangles in the outer ring indicate clusters of hits from contigs annotated to be eukaryotes and represent putative eukaryotic radiations. Black trapezoid shapes indicate the two branches containing giant viruses and bacterial hosts.

To assess the diversity of Fz, we searched for Fz proteins from TnpB and Fz seeds using structural mining of an AlphaFold database and sequence profile mining of the non-redundant NCBI database (Methods) and then built a phylogenetic tree from 3,003 curated representatives (Fig. 1a). The tree contained many branches with prokaryotic TnpB proteins, 80 viral proteins and 649 proteins from various eukaryotic species (Extended Data Fig. 1 and Supplementary Table 1). Eukaryotic hits were mainly spread in two distinct large branches that reflected the two types of Fz protein, Fz1 and Fz2, that have been previously described^7^. The branches forming Fz1 and Fz2 emerged from two different branches of TnpBs, indicating potential independent origins whereby two distinct TnpBs were horizontally transferred to eukaryotic hosts. Fz1 is highly spread in fungi, particularly in incertae sedis species; however, it is also found in protists, arthropods, plants and eukaryotic viruses, in particular giant viruses. Fz2 is found broadly in fungi and in a few instances in molluscs, choanoflagellates and eukaryotic viruses, most of which are also giant viruses. TnpB has been observed in both Fz branches, with a substantial presence in branches hosting giant viruses that infect hosts living in symbiosis with bacteria (for instance, *Acanthamoeba castellanii mamavirus*), and sporadically in branches hosting SAR (Stramenopiles, Alveolates and Rhizaria) or fungi, raising the possibility that TnpBs were horizontally transferred from prokaryotes to eukaryotic hosts (Supplementary Table 1). Although the two Fz systems probably emerged from the transfer of two distinct TnpBs to two eukaryotic hosts, the diversity of eukaryotic hosts for both Fz systems and the presence of Fz in eukaryotic viruses and in numerous fungi, both of which are potential vectors for horizontal gene transfer, indicate that Fz is also likely to have been transferred among eukaryotic species. In addition to the Fz1 and Fz2 branches, we found branches and sometimes single leaves with eukaryotic proteins emerging from other diverse TnpB branches from around the tree. Manual examination of these eukaryotic radiations showed that they were from hosts featuring lifestyles deeply connected to bacterial species (for instance, bacterivores or living with parasitic bacteria; Supplementary Table 1). These examples further indicate potential continuing acquisition of TnpB from bacteria to generate eukaryotic Fzs.

## Structural architecture of Fz and TnpB

**Extended Data Fig. 2:**
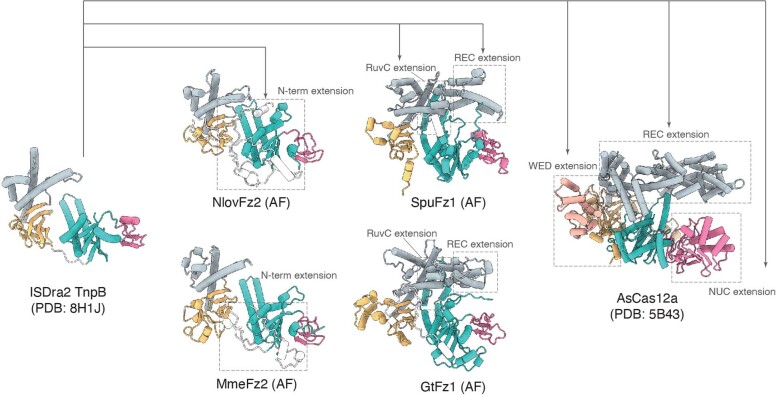
Structural overview and comparative analysis of representative Fanzor proteins. Structural comparison of ISDra2 TnpB (PDB: 8H1J), NlovFz2 (AlphaFold model, AF), MmeFz2 (AF), SpuFz1 (AF), GtFz1 (AF) and AsCas12a (PDB: 5B43). Color coding represents common structural regions. Arrows highlight the hypothesized evolutionary progression from TnpB to Fanzors and Cas12a. Fanzor1, Fanzor2 and Cas12a likely emerged independently from TnpBs and acquired various extensions in the N-terminal region (N-term), REC domain, RuvC domain and NUC domain. The extensions in Fanzor1 (represented by SpuFz1 and GtFz1) involve the REC and RuvC domains, which form a channel that is similar to the one found in Cas12a.

We next compared the structural architectures of TnpB from the IS200/IS605 transposon family of *Deinococcus radiodurans* (ISDra2 TnpB)^8^, Cas12a from *Acidamanococcus* sp. (AsCas12a)^9^, a relative of TnpB from CRISPR–Cas class 2 type V-A systems, two Fz1 orthologues from the soil fungus *Spizellomyces punctatus* (SpuFz1) and the algae *Guillardia theta* (GtFz1), and two Fz2 orthologues from Percolozoa *Naegleria lovaniensis* (NlovFz2) and a multicellular eukaryote, the marine mollusc *Mercenaria mercenaria* (MmeFz2) (Fig. 1b). Despite the strong divergence in sequence and size among these systems, we found that they shared a similar core domain architecture that included a WED region and a RuvC region. Fz encompasses a RuvC domain, which has a predicted active catalytic site formed by positively charged residues. This site is found in the comprehensive RuvC region in proteins such as AsCas12a, ISDra2 TnpB, SpuFz1, GtFz1, NlovFz2 and MmeFz2. The core regions of these proteins have various insertions that are specific to each family. The largest of these proteins, AsCas12a, has an approximately 900-amino-acid (aa) insertion in the WED region, known as the REC region, which forms a channel that protects the spacer–target hybrid region and is likely to be involved in R-loop formation^10^. In ISDra2 TnpB, NlovFz2 and MmeFz2, this REC region is reduced to three helices (approximately 100 aa), which probably serve as a minimal structure to achieve the same function. SpuFz1 has these three helices and a further insertion of 150 aa, which forms a globular extension that interacts with another extension inserted in the RuvC domain, contributing to a channel shape that is similar to (although smaller than) the REC region of AsCas12a (Extended Data Fig. 2). Although NlovFz2 and MmeFz2 bear resemblances to ISDra2 TnpB, each harbours a unique amino-terminal disordered region, with NlovFz2 featuring a 96-aa segment and MmeFz2 having a 61-aa segment. The structural differences among ISDra2 TnpB, Fz1 and Fz2 indicate potential selection of distinct features related to the mechanisms and/or functions of these systems, but the conservation of the core region and predicted active catalytic sites indicates that Fz may be able to perform RNA-guided targeting.

## Identification of ωRNA from *Fz* loci

**Extended Data Fig. 3:**
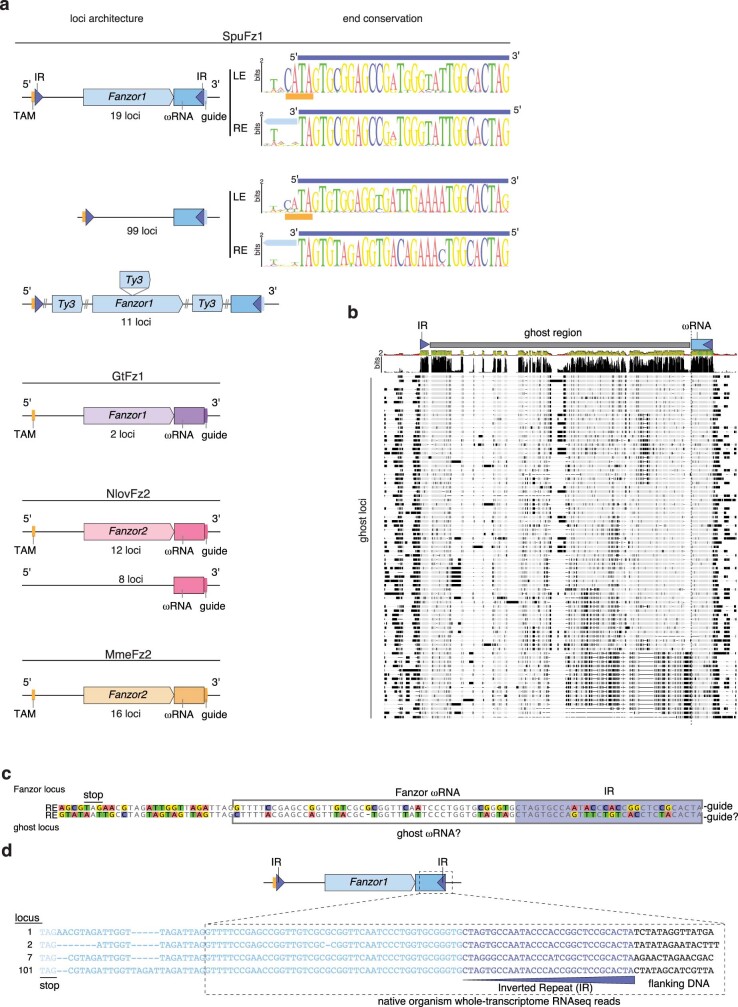
Fanzor and standalone ghost loci architecture. **a**, Top: Comparison of loci architecture for *Fz* gene and ghost in *S. punctatus* and comparison of their Weblogo inverted repeat sequences (IR). IRs are shown as blue triangles, TAM regions as orange rectangles, *Fz* gene as a light blue arrow, ωRNA regions as medium blue rectangles with a downstream light blue rectangle showing the guide (spacer region). Fanzor and ghost loci share similar but distinct IRs. Bottom: Comparison of loci architecture for *Fz* gene and ghost in *G. theta*, *N. lovaniensis*, and *M. mercenaria*. **b**, Sequences alignments of ghost loci from IR to IR. Schematic of the architecture is shown on top of the alignment. Conservation is shown as bits on the top row. In the alignment, grey color indicates identity, black color indicates differences and lines indicate gaps. The sequences are sorted according to a phylogenetic tree made from the full nucleotide sequences in FastTree. IRs and ωRNA regions are strongly conserved across all ghost loci. **c**, Sequence alignment of the ωRNA region or IR of a Fanzor locus and a ghost locus. Nucleotide background colors highlight differences between ωRNAs. **d**, Small RNA-seq of Fanzor loci from *S. punctatus* shows expression of associated ωRNAs.

**Extended Data Fig. 4:**
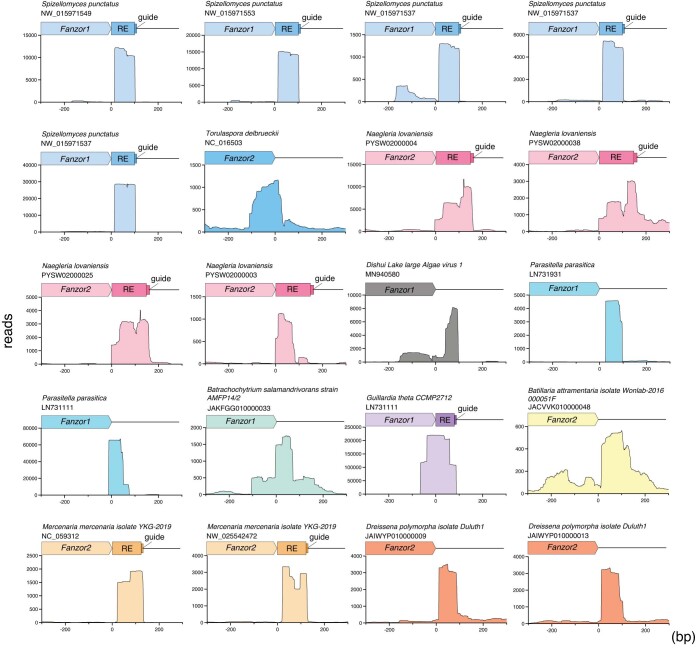
Small RNA-seq of RNPs of Fz orthologs expressed in *Saccharomyces cerevisiae*. Small RNA-seq of RNPs of Fz orthologs expressed in *S. cerevisiae* mapped to the *Fz* loci. RE, transposon right end.

To test the RNA-guided endonuclease activity of Fz, we focused on SpuFz1, given its larger REC domain relative to Fz2. SpuFz1 is encoded in a roughly 2.1-kilobase pair (kbp) locus containing a single open reading frame (ORF) flanked by well-conserved, non-degraded transposon inverted repeat (IR) structures (left-end and right-end). In the sequenced genome of *S. punctatus* DAOM BR117, we identified 42 loci containing full-length (19) or partial *Fz* genes and 134 loci containing two regions homologous to the IRs surrounding the *Fz* gene but lacking the *Fz* ORF (we refer to the latter hereafter as ghost *Spu-1* elements) (Extended Data Fig. 3a,b and Supplementary Data 1 and 2). SpuFz1 proteins are extremely well conserved, sharing around 82% sequence identity, including the catalytic sites, which indicates recent duplication or strong selection. The complete *Fz* genes are surrounded by 30-nucleotide (nt) IRs with two further conserved nts, CA, upstream of the 5′ (also referred to as left-end^6^) IR (Extended Data Fig. 3a). We identified 11 *Fz* loci that contained further genes embedded by the IRs, including Gypsy/Ty3 inserted upstream or downstream of or in the *Fz* gene, although the latter is indicative of a possible past transposition of Ty3 instead of an association with *Fz* (Extended Data Fig. 3a). The ghost loci encode a conserved region of around 550 nt flanked by 30-nt conserved IRs, although the sequence differs from that of the *Fz* loci IRs at seven positions (Extended Data Fig. 3a). Ghost loci also have the conserved CA motif upstream of the 5′ IR (Extended Data Fig. 3a). The 75-nt region encompassing the 3′ (also referred to as right-end) IR has similarity to the region in *Fz* loci found between the stop codon and the 3′ IR (Extended Data Fig. 3c). To determine whether the 80-nt region in *Fz* loci encoded an ncRNA, we performed small RNA sequencing (RNA-seq) on *S. punctatus*. The results showed expression of an 88–90-nt ncRNA species downstream of *Fz* in four of these loci (Extended Data Fig. 3d). We observed that the transcripts consistently extended 14–15 nt beyond the conserved 75-nt region at the 3′ end, and that these extensions contained variable sequences. The conserved ncRNA spanning the IR and the variable extension is indicative of a possible ωRNA with a 75-nt scaffold and 14–15-nt guide region. To confirm the interaction between SpuFz1 and the ncRNA, we heterologously expressed 10xHis-maltose-binding protein (MBP)-tagged SpuFz1 with downstream IRs of representative *Spu-1* elements in *Saccharomyces cerevisiae* and performed pull-down experiments followed by small RNA-seq. We found that the ribonucleoprotein (RNP) complex contained the same ncRNA species observed in the native organism, indicating that SpuFz1 binds to the ncRNA transcribed from the 3′ IR and indicating it is an ωRNA (Fig. 1c). The ωRNA coding sequence of GtFz1 overlapped with 17 aa of the carboxy-terminal protein-coding sequence, similar to (although smaller than) the overlap observed for the ωRNA of ISDra2 TnpB^8,11^. Using the same workflow, we confirmed the expression of the ωRNA for 20 *Fz* loci from ten organisms (Extended Data Fig. 4 and Supplementary Tables 2 and 3), including an *Fz1* locus from *G. theta*, four *Fz2* loci from *N. lovaniensis*, and two *Fz2* loci from *M. mercenaria* (Fig. 1c and Extended Data Fig. 4). Secondary structure prediction of these ωRNAs revealed a stem–loop structure that contained the flanking DNA sequence (Fig. 1c).

## DNA cleavage in vitro by Fz-ωRNA RNP

**Fig. 2:**
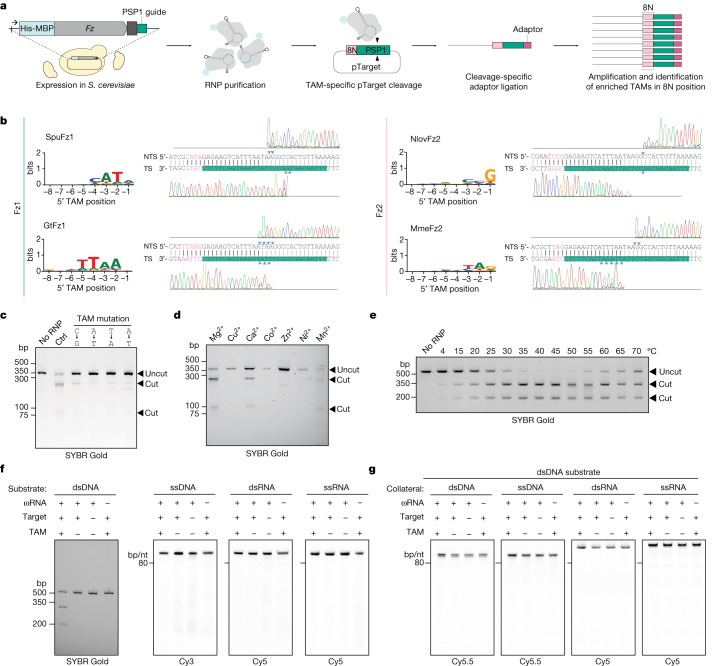
Biochemical characterization of Fz. **a**, Scheme of TAM identification screen in *S. cerevisiae*. **b**, TAMs of four representative Fz orthologues (SpuFz1, GtFz1, NlovFz2 and MmeFz2) and Sanger sequencing traces of the dsDNA targets with PSP1 target sequence matching reprogrammed ωRNA guides. The non-templated addition of a final base was an artefact of the polymerase (as a terminal A in the target strand trace and a terminal T in the NTS trace). Cleavage sites are indicated by blue triangles. **c**, SpuFz1-mediated target dsDNA cleavage with TAM mutations. Target dsDNA substrates were column-purified after proteinase treatment and run on a 2% agarose gel. **d**, SpuFz1-mediated target dsDNA cleavage dependence on divalent metal ions. Target dsDNA substrates were column-purified after proteinase treatment and run on a 2% agarose gel. All experiments except those corresponding to this panel were performed with Mg^2+^. **e**, Temperature dependence of SpuFz1-mediated target dsDNA cleavage activity. All experiments except those corresponding to this panel were performed at 37 °C. **f**, SpuFz1 cleaves only target dsDNA. Target nucleic acid species were column-purified after proteinase treatment and run on a 2% agarose gel (for dsDNA) or denaturing polyacrylamide gel (for ssDNA, dsRNA and ssRNA). The gels were imaged with SYBR Gold (for dsDNA channels) or Cy3 (for ssDNA channels) and Cy5 (for dsRNA and ssRNA channels). **g**, SpuFz1 did not show collateral activity on Cy5.5-labelled collateral dsDNA, Cy5.5-labelled collateral ssDNA, Cy5-labelled collateral dsRNA or Cy5-labelled collateral ssRNA. Representative gel images from three independent technical replicates are shown. Ctrl, control; TS, target strand.

On the basis of its structural similarity to TnpB and Cas12, we proposed that Fz could perform DNA cleavage, with the 3′-terminal flanking sequence of the ωRNA functioning as a guide sequence to direct Fz to its target. To test this hypothesis, we adopted the previously developed target-adjacent motif (TAM) identification assay used for OMEGA systems^4^. The 3′-terminal ωRNA flanking sequence in the *S. cerevisiae* expression vector for four Fz orthologues (SpuFz1, GtFz1, NlovFz2 and MmeFz2) was replaced by a 30-nt sequence (PSP1 guide) matching a target site adjacent to an 8-base pair (bp) (8N) randomized region in a plasmid library. Fz RNP complexes were purified from yeast and used for plasmid library cleavage assays (Fig. 2a). Deep sequencing of the cleaved products revealed enrichment of specific 5′ sequences in the randomized 8N region upstream of the target sequence (5′-CATA-TAM sequence for SpuFz1, 5′-TTAAN for GtFz1, 5′-CCG for NlovFz2 and 5′-TAG for MmeFz2) (Fig. 2b). Sanger sequencing of the cleaved products revealed unique cleavage patterns for each orthologue (Fig. 2b). SpuFz1 cleaved after the 16th and 17th bases on the non-target strand (NTS) and after the 20th and 21st bases on the target strand, generating 5′ overhangs. GtFz1 cleaved after the 14–17th bases on the NTS and after the 14–16th bases on the target strand, which could generate both sticky and blunt ends. NlovFz2 cleaved after the 19th base on both the NTS and target strand, generating blunt ends. Finally, MmeFz2 cleaved after the 17th and 18th bases on the NTS and after the 9–14th bases on the target strand, generating 3′ overhangs. Collectively, these results demonstrate that Fz1 and Fz2 are ωRNA-guided endonucleases.

We further characterized Fz1 using recombinant SpuFz1 RNP from *S. cerevisiae*, confirming that mutating any base in the TAM sequence completely abolished cleavage, indicating a strong dependence on the TAM for RNA-guided DNA endonuclease activity (Fig. 2c). Cleavage activity of SpuFz1 was supported by magnesium, calcium and manganese (Fig. 2d) and had a broad temperature range (from 4 °C to 70 °C), consistent with the mesophilic temperature habitat of the host organism *S. punctatus* (Fig. 2e). We also tested the activity of SpuFz1 on other types of substrate (single-stranded DNA (ssDNA), double-stranded RNA (dsRNA) and single-stranded RNA (ssRNA)) and found that SpuFz1 performed ωRNA-guided, TAM-dependent and target-dependent double-stranded DNA (dsDNA) cleavage but did not cleave targeted ssDNA (Fig. 2f). We further confirmed that SpuFz1 did not have any robust cleavage activity on collateral dsDNA, ssDNA, dsRNA or ssRNA substrates on dsDNA target recognition (Fig. 2g).

## Fz functions as a human genome editor

**Fig. 3:**
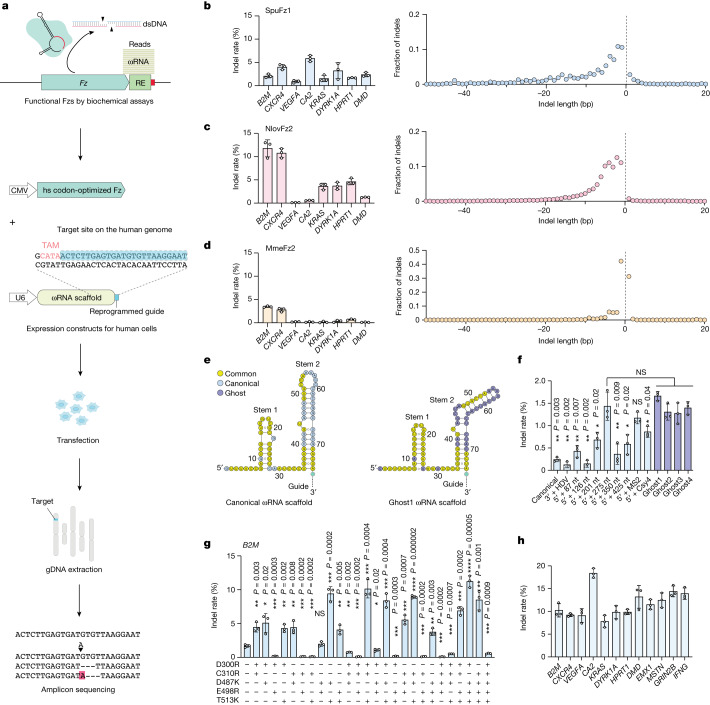
Human genome engineering with Fz. **a**, Workflow for testing Fz activity in HEK293FT cells. **b**–**d**, Indel rates and average indel lengths generated by SpuFz1 (**b**), NlovFz2 (**c**) and MmeFz2 (**d**) at eight genomic loci in HEK293FT cells. Left, average indel rate (%); data are presented as mean ± s.d. (*n* = 3). Right, average indel length at *B2M* target site. **e**, Secondary structure prediction of canonical (left) and ghost (right) ωRNAs for SpuFz1. Identical nucleotides between canonical and ghost ωRNA are highlighted in yellow. Guide region has been abbreviated for visualization purposes. **f**, SpuFz1 activity at *B2M* with canonical ωRNA, modified ωRNA and ghost ωRNA scaffolds. Average indel rate (%); data are presented as mean ± s.d. (*n* = 3). Statistical analysis was by two-tailed *t* test. *P < 0.05; **P < 0.01. **g**, Indel activity of combinatorial SpuFz1 point mutants at *B2M*. Average indel rate (%); data are presented as mean ± s.d. (*n* = 3). Statistical analysis was by two-tailed *t* test. *P < 0.05; **P < 0.01; ***P < 0.001; ****P < 0.0001. **h**, SpuFz1-v2 activity at 12 human genomic loci. Average indel (%); data are presented as mean ± s.d. (*n* = 3). gDNA, genomic DNA; HDV, hepatitis delta virus; hs, *Homo sapiens*; NS, not significant.

**Extended Data Fig.5:**
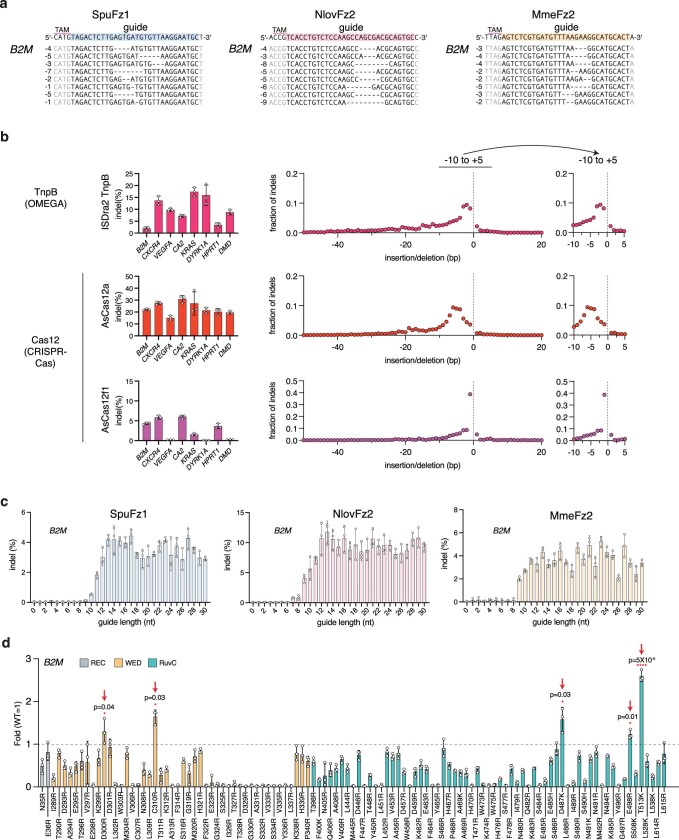
Human genome targeting activity of Fanzor, TnpB and Cas12. **a**, Indels generated by Fzs at the *B2M* locus ordered by abundance, with indel size at left. Left: SpuFz1. Middle: NlovFz2. Right: MmeFz2. **b**, Indel rates and average indel length generated by ISDra2 TnpB, AsCas12a and AsCas12f1 at 8 genomic loci in HEK293FT cells. Left: The average indel (%) generated is shown with an error bar showing standard deviation (n = 3). Right: Indel pattern from −50 to +20 bp with inset showing the indel pattern spanning 10-bp deletion to 5-bp insertion. **c**, Targeting SpuFz1, NlovFz2 and MmeFz2 to a representative *B2M* locus in HEK293FT cells with ωRNAs containing guides of various lengths. The average indel (%) generated is shown with an error bar showing standard deviation (n = 3). Left: SpuFz1. Middle: NlovFz2. Right: MmeFz2. **d**, Indel activity (relative to WT) of 111 single point mutants measured in HEK293T cells at a representative *B2M* locus. Red arrows indicate the five mutations tested further in a combinatorial manner. The average indel (%) generated is shown with an error bar showing standard deviation (n = 3). Statistical analysis was performed using a two-tailed t-test. Significant increase compared to WT is indicated by (*). *, p < 0.05; ****, p < 0.0001.

Next, we tested whether the Fz OMEGA system could be harnessed to achieve RNA-guided DNA cleavage in the genome of human cells. We focused on four orthologues (SpuFz1, GtFz1, NlovFz2 and MmeFz2) with in vitro activity and tested their activities in human cells. A plasmid encoding the human-codon-optimized Fz protein and the U6 promoter-driven ωRNA expression plasmid were transiently transfected into HEK293FT cells. After 72 h, genomic DNA was extracted and analysed by deep sequencing for the presence of insertions and deletions (indels) at the targeted sites (Fig. 3a). To assess the programmability of the system, we screened eight guides targeting eight different loci: *B2M*, *CXCR4*, *VEGFA*, *CA2*, *KRAS*, *DYRK1A*, *HPRT1* and *DMD*, with ISDra2 TnpB^8^, AsCas12a^12^ and AsCas12f1 (a minimal editor of Cas12 lineage)^13^ as benchmarks. We confirmed that SpuFz1, NlovFz2 and MmeFz2 induced indels at these sites with varying efficiency, up to 11.8% by NlovFz2, whereas GtFz1 did not induce detectable indels (0.01%) at these eight different loci (Fig. 3b–d and Extended Data Fig. 5a). The overall editing efficiency of the three wild-type Fz proteins was comparable with that of wild-type AsCas12f1 (Extended Data Fig. 5b). Deep sequencing of the target loci revealed that these three orthologues had different indel patterns (Fig. 3b–d). SpuFz1 and NlovFz2 caused larger deletion patterns, closer to the pattern observed for AsCas12a than that of SpCas9 (ref. ^14^). ISDra2 TnpB also caused a larger deletion pattern (Extended Data Fig. 5b). MmeFz2 uniquely showed a prominent 1-bp deletion pattern, similar to that of AsCas12f1 (Extended Data Fig. 5b). To further characterize the activity of these three Fz orthologues, we tested a range of guide lengths targeting a representative locus, *B2M*, on the human genome. SpuFz1, NlovFz2 and MmeFz2 showed minimal activity with 10-nt, 7-nt and 9-nt guides, respectively, and the efficiency increased with increasing guide length, reaching a plateau around 13-nt, 12-nt and 11-nt guides, respectively (Extended Data Fig. 5c). The native guide lengths identified by RNP RNA-seq (14-nt, 21-nt and 11-nt) were within the plateaus.

On the basis of the structural predictions and the minimal guide length requirement in human cells, we focused on SpuFz1 for optimization for activity in human cells. We first tested whether extending or modifying the ωRNA scaffold improved activity. We tested extensions from 87 nt to 425 nt and found that the 275-nt extension of the 5′ sequence or addition of an MS2 stem–loop to the 5′ of the ωRNA scaffold improved the activity of SpuFz1. We also assessed the function of ghost ωRNAs, which lack a *Fz* gene in their loci. RNA structure prediction showed that ghost ωRNAs had a distinct stem 2 structure with a 3-nt bulge instead of an 8-nt hairpin loop (Fig. 3e). The 75-nt ghost ωRNAs from four different loci achieved indel efficiency comparable with that of the 5′ MS2 stem–loop or 5′ extended canonical ωRNA from the original SpuFz1 locus (Fig. 3f). On the basis of these results, we used the 5′ 275-nt extended canonical ωRNA for downstream experiments. To further improve activity, we sought to increase the binding strength of SpuFz1 proteins to ωRNA and target DNA by mutating residues selected on the basis of the structural model to arginine, lysine or histidine. We tested the activity of 111 SpuFz1 single point mutants at the *B2M* target site (Extended Data Fig. 5d). We identified five mutants (D300R, C310R, D487K, E498R and T513K) that showed increased indel efficiency compared with the wild-type SpuFz1 (Extended Data Fig. 5d). By combining these mutations, we further boosted efficiency (Fig. 3g). We selected one combination, C310R/D487K/T513K (hereafter referred to as SpuFz1-v2) and confirmed that it showed improved gene editing efficiency at eight genomic sites (Fig. 3h). Collectively, these data demonstrate the potential of Fz for human genome engineering applications.

## Structure of SpuFz1–ωRNA–target DNA complex

**Fig. 4:**
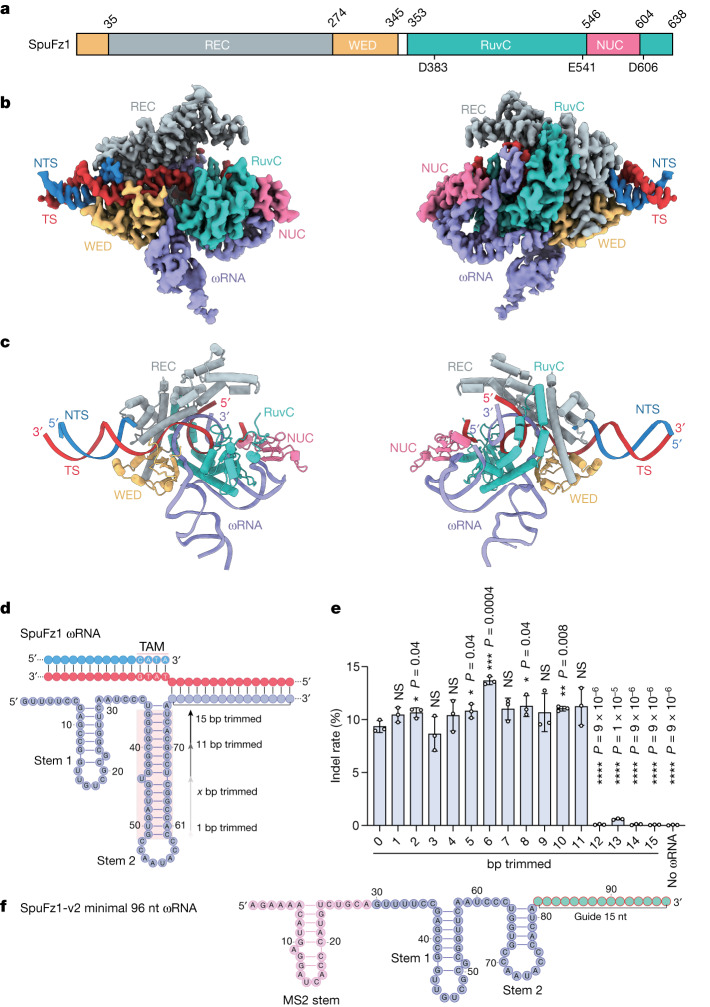
Structure of SpuFz1. **a**, Domain organization of SpuFz1. White regions represent the flexible loop. **b**, Cryo-EM map of SpuFz1–ωRNA–target DNA complex. **c**, Structural model of SpuFz1–ωRNA–target DNA complex. REC domain is coloured grey, WED domain is coloured yellow, RuvC domain is coloured light blue, NUC domain is coloured pink, ωRNA is coloured purple, DNA TS is coloured red and DNA NTS is coloured blue. **d**, Diagram of SpuFz1 ωRNA and trimmed variants. **e**, SpuFz1-v2 activity at *B2M* with trimmed ωRNA variants. Average indel rate (%); data are presented as mean ± s.d. (*n* = 3). Statistical analysis was by two-tailed *t* test. *P < 0.05; **P < 0.01; ***P < 0.001; ****P < 0.0001. **f**, Minimal SpuFz1 ωRNA design.

**Fig. 5:**
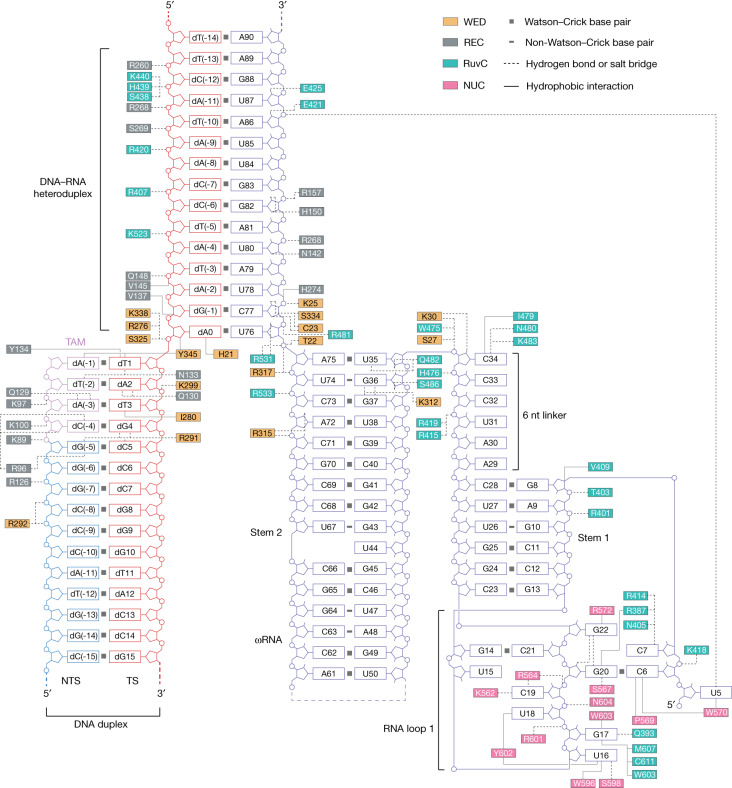
Schematic of ωRNA and target DNA recognition. The aa residues that engage in interactions with nucleic acids are highlighted in coloured boxes, with the colours specified by the domains where these residues reside. Hydrogen bonds and salt bridges are shown by dashed lines. Hydrophobic interactions are shown by solid lines.

**Extended Data Fig. 6:**
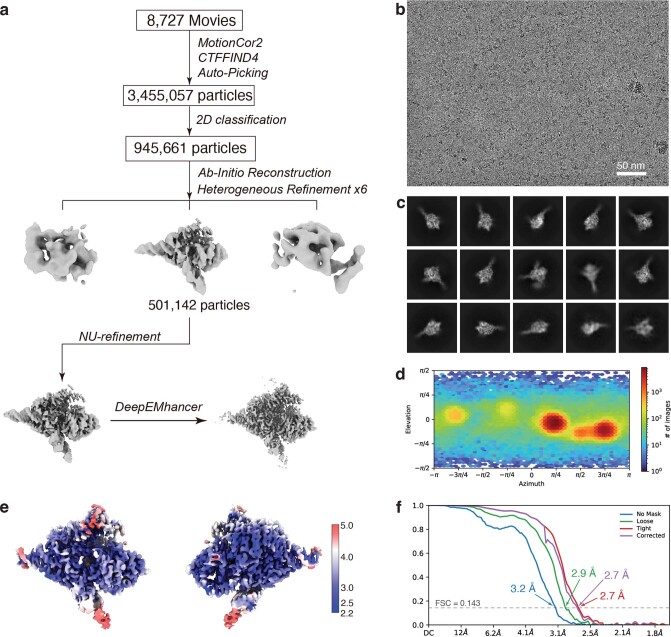
Cryo-EM data processing for the SpuFz1-ωRNA-target DNA complex. **a**, Flow chart of cryo-EM data analysis. **b**, Representative cryo-EM image from 8,727 movies. **c**, Representative and 2D averages. **d**, Angular distribution of the SpuFz1-ωRNA-target DNA particles in the final round of 3D refinement. **e**, Sharpened EM density maps colored by local resolution as calculated by CryoSPARC. **f**, The ‘gold-standard’ FSC curves of the SpuFz1-ωRNA-target DNA complex.

**Extended Data Fig. 7:**
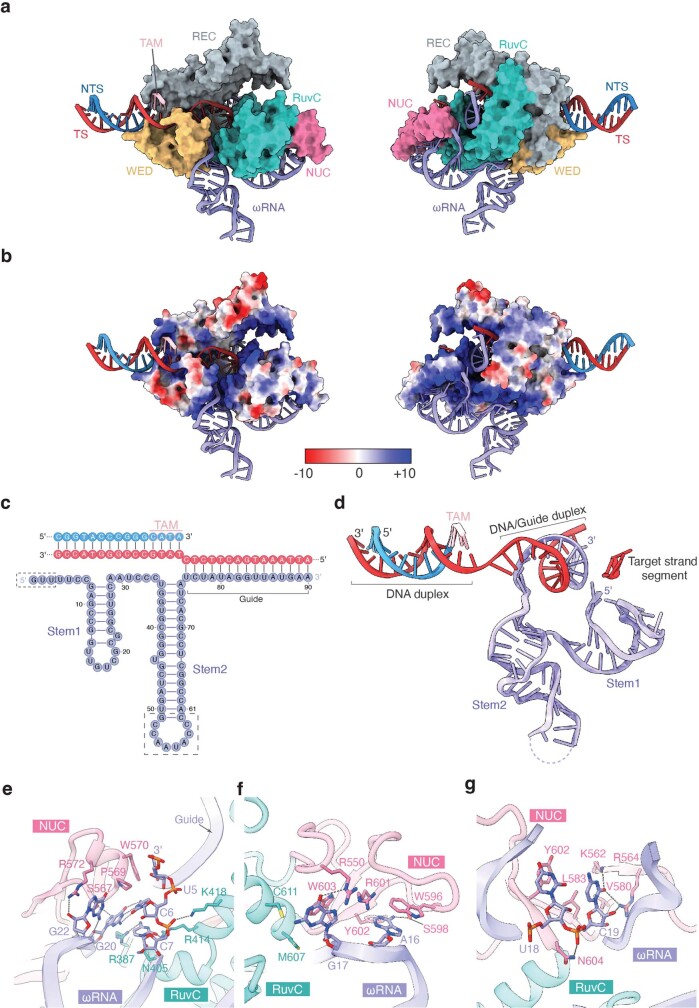
Structure of the ωRNA and target DNA recognition. **a**, The overall structure of the SpuFz1-ωRNA-target DNA complex. Domain structure shown in surface and by colors. **b**, Electrostatic surface potential of SpuFz1. **c**, Schematic of the ωRNA and target DNA. Disordered regions are enclosed in a dashed box. **d**, Structural model of the ωRNA and target DNA. **e**-**g**, The structural details of the interaction between stem loop 1 and SpuFz1.

To understand the DNA cleavage mechanism of SpuFz1, we determined the cryogenic electron microscopy (cryo-EM) structure of the full-length wild-type SpuFz1 (residues 1–638) in complex with the native ωRNA, a 54-nt target DNA strand and a 24-nt non-target DNA strand at a resolution of 2.7 Å (Figs. 4 and 5 and Extended Data Fig. 6). EM densities were sufficient to construct most of the models for SpuFz1, ωRNA and target DNA. The structure showed that SpuFz1 adopts a bilobal architecture, comprising a recognition (REC) lobe and a nuclease (NUC) lobe. The REC lobe consists of the REC domain and WED domain, and the NUC lobe is composed of the RuvC domain and the NUC domain (Fig. 4b,c). The DNA duplex containing the TAM sequence is surrounded by the REC and WED domains (Extended Data Fig. 7a). The heteroduplex of ωRNA and target DNA is accommodated by the positively charged channel formed by the REC domain and the RuvC domain (Extended Data Fig. 7b).

The ωRNA structure of SpuFz1 includes a 15-nt guide segment and a 75-nt RNA scaffold. The RNA scaffold is composed of two stems and a linker (Fig. 5 and Extended Data Fig. 7c,d). The scaffold of the ωRNA is recognized by the positively charged surface formed by the WED, RuvC and NUC domains of SpuFz1 (Extended Data Fig. 7b). In the structure, the first three nucleotides, U5, C6 and C7, of the ωRNA scaffold form extensive interactions with SpuFz1, where the base of U5 is sandwiched by the NUC domain and guide region of the ωRNA, forming a stacking interaction with W570 of the NUC domain and a hydrogen bond with the backbone phosphate of A86. The second nucleotide, C6, forms a base pair with G20, a nucleotide of stem–loop 1. The third nucleotide, C7, forms hydrogen bonds with R414 and R387 of the RuvC domain (Fig. 5 and Extended Data Fig. 7e). The nucleotides from stem–loop 1 (U16, G17, U18, C19, G20 and G22) extensively interact with the residues of the RuvC and NUC domains, including W596^NUC^, R601^NUC^, N604^NUC^, S598^NUC^, Y602^NUC^, R550^NUC^, C611^RuvC^, M607^RuvC^, W603^NUC^, L583^NUC^, K562^NUC^, R564^NUC^, S567^NUC^ and R572^NUC^ (Fig. 5 and Extended Data Fig. 7e–g). The 6-nt linker between stem 1 and stem 2, A29 to C34, interacts with the RuvC domain, with the side-chain residues of R415 and R419 interacting with the backbone phosphate of C32, whereas the side chains of residues K483 and N480 interact with the base group of C34. Stem 2 is mainly recognized by the WED and RuvC domains: the phosphates of A72 and A75 interact with R315 and R317 of the WED domain, and the base groups of U35 and G37 interact with Q482 of the RuvC and K312 of the WED domain, respectively. The core-distal part of stem 2 (C40–G70) does not contact SpuFz1, and the structure of this region is flexible, as reflected by the low local resolution and lack of EM density in the loop region of stem 2 (Extended Data Fig. 6e). This observation indicated that this region of stem 2 could be omitted, making a more compact ωRNA for applications. To test this hypothesis, we systematically trimmed the stem 2 region and confirmed that the C40–G70 region is not essential for activity of SpuFz1 in human cells (Fig. 4d,e), giving a final ωRNA design for 96 nt (29-nt MS2 stem–loop + 52-nt trimmed ωRNA scaffold + 15-nt guide) (Fig. 4f).

Fifteen bp of the RNA–DNA heteroduplex are visible in the structure, positioned within a positively charged channel created by the WED, REC and RuvC domains (Extended Data Fig. 7a–d). The first complementary pairing between dA0 in the DNA and U76 in the RNA is stacked with the WED domain. Polar interactions are formed by T22 of the WED domain and R531 of the RuvC domain with U76 in RNA, and stacking interactions are formed by H21 of the WED domain and dA0 in the DNA, facilitating heteroduplex formation. Sugar–phosphate backbone interactions are formed between C77, U78, A81 and G83 in the RNA and residues R481^RuvC^, K25^WED^, R268^REC^ and R157^REC^ of SpuFz1. The interactions formed by the sugar–phosphate backbone interactions were also observed between the DNA (dA(-2), dC(-6), dA(-8), dA(-9), dA(-10), dA(-11), dC(-12)) and protein (Q148^REC^, R407^RuvC^, R420^RuvC^, S269^REC^, R268^REC^, K440^RuvC^, R260^REC^) (Fig. 5).

**Extended Data Fig. 8:**
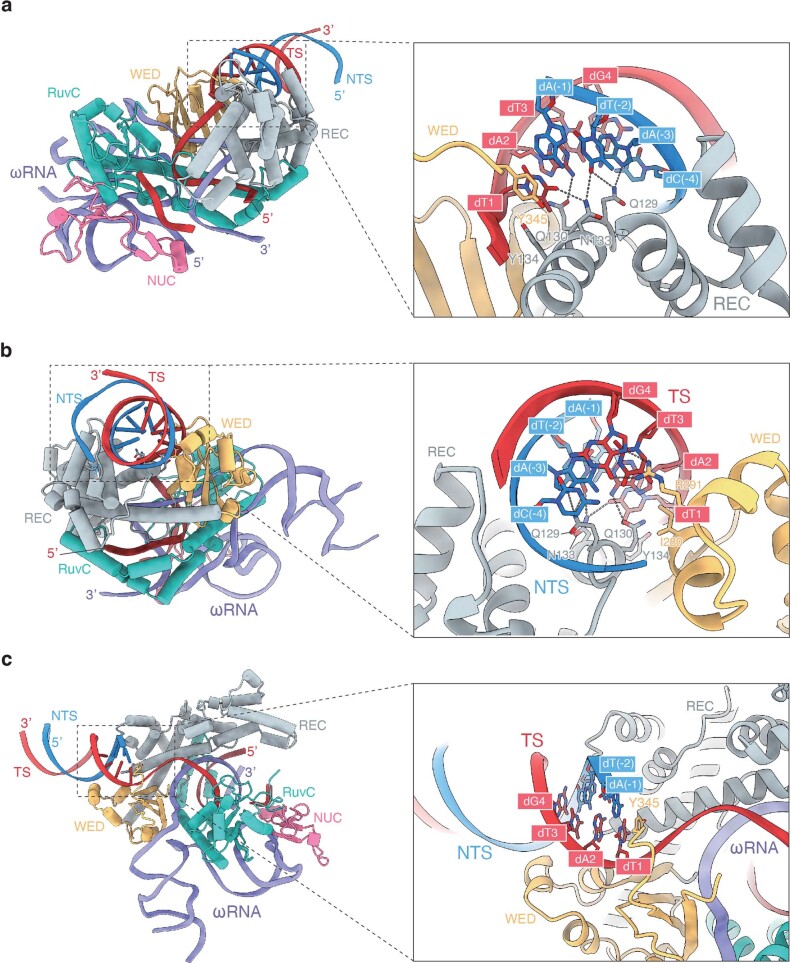
TAM recognition by SpuFz1. **a**-**b**, Interactions between the TAM and SpuFz1. **c**, Interactions between the end of the TAM and the WED domain loop of SpuFz1.

The DNA TAM region is recognized by the REC and WED domains. The NTS bases of the 5′-CATA-3′ TAM interact with the REC domain, whereas the target strand bases interact with both the REC and WED domains. Specifically, hydrogen bonds are formed by dC(-4), dA(-3) and dT(-2) on the NTS with residues R96, Q129 and N133 of the REC domain and by dG4, dA2 and dT1 on the target strand with residues R291 of the WED domain, Q130 of the REC domain and N133 of the REC domain, respectively (Fig. 5 and Extended Data Fig. 8a,b). In addition, base dT1 on the TAM interacts with Y345 in the loop of the WED domain, which is the starting point for DNA unwinding (Extended Data Fig. 8c). These results show the recognition mechanism of the SpuFz1 5′-CATA-3′ TAM.

**Extended Data Fig. 9:**
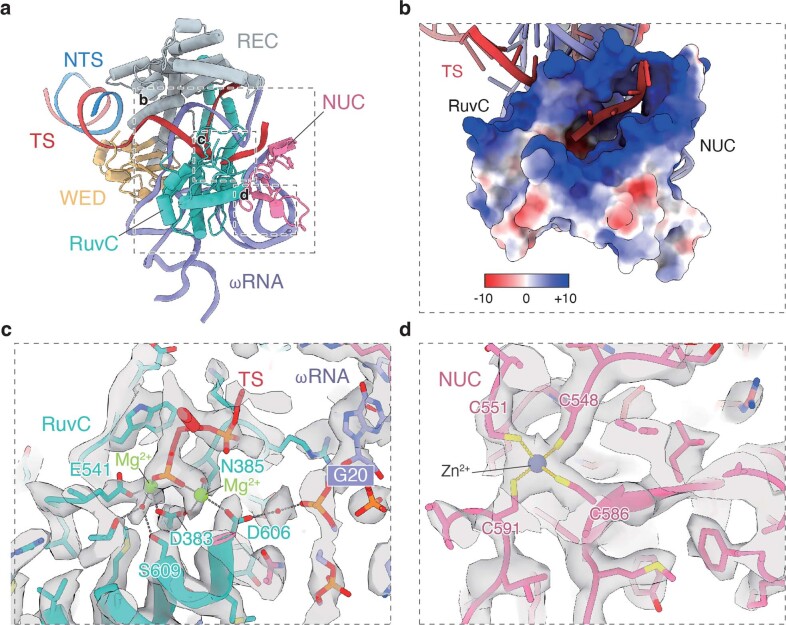
The structure of RuvC and NUC domains and the active site of SpuFz1. **a**, Structure of a DNA target strand segment bound to the RuvC and NUC domains of SpuFz1. **b**, Electrostatic surface potential of the RuvC and NUC domains. **c**, Structural details of the active site. **d**, Structure of the zinc finger motif in the NUC domain of SpuFz1.

We observed in the structure that a downstream segment of the target strand bound to the positively charged channel formed by the RuvC and NUC domains (Extended Data Fig. 9). Two magnesium ions were coordinated by residues E541, D383, N385, and D606 and by the terminal nucleotide phosphate backbone of the target strand. Notably, a putative water molecule was coordinated by the catalytic residue D606 and the G20 phosphate backbone of the ωRNA (Extended Data Fig. 9c). The interactions of D606 with the ωRNA through the putative water molecule indicate that the active site is stabilized by the ωRNA and indicate that the ωRNA may have a role in the catalytic function of the protein. These results provide insight about the cleavage mechanism of SpuFz1, which potentially involves the ωRNA.

## Discussion

RNA-guided systems couple programmable nucleic acid recognition with enzymatic activity, enabling a single protein or protein complex to target multiple sites. For example, in prokaryotes, the RNA-guided CRISPR–Cas system provides adaptive immunity against a range of invading foreign genetic elements. Although eukaryotes also use this coupling strategy, such as in small interfering RNA and microRNA-mediated gene regulation, mechanistically, these systems are distinct from prokaryotic systems. The recently described OMEGA systems seem to combine RNA-guided recognition with transposition of mobile elements^4,15^. Here, we showed that the eukaryotic Fz protein, which shares remote homology with the OMEGA effector TnpB, is an RNA-guided endonuclease, demonstrating a universal RNA-guided mechanism that spans all kingdoms of life.

**Fig. 6:**
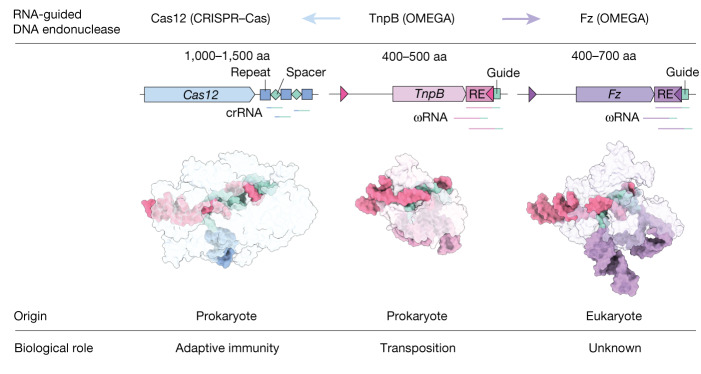
Fz, TnpB and Cas12. OMEGA systems are the ancestors of CRISPR–Cas systems. The ancestral ωprotein TnpB became associated with CRISPR arrays and evolved into Cas12 in prokaryotes and Fz in eukaryotes. Cas12 works as a CRISPR effector protein for adaptive immunity. TnpB helps to propagate insertion sequences in which it is encoded. The biological roles of Fzs remain unknown. ωproteins Fz and TnpB are relatively compact proteins (400–700 and 400–500 aa, respectively) compared with Cas12 proteins (1,000–1,500 aa). crRNA, CRISPR RNA.

Distinct TnpBs gave rise to both Cas12s and Fzs, with Cas12 evolving for adaptive immune function and guided transposition in prokaryotes^14,16^, whereas Fzs became adapted for function in eukaryotes (Fig. 6). Multiple horizontal gene transfer events led to the formation of Fz1 and Fz2 from diverse TnpBs, and continuing transfers seem to also occur, as indicated by the presence of prokaryotic–eukaryotic symbiotic hosts (and the presence of TnpB in Fz branches). Fz also seems to have propagated among eukaryotes, probably via eukaryotic viruses (Extended Data Fig. 1) and eukaryotic symbionts. For example, we found Fzs in viruses with potential algae or mollusc hosts, both of which contain Fzs (for instance, GtFz1 and MmeFz2) (Supplementary Table 1). Similar to TnpB and some Cas12 family members, we found that *Fz1* from the fungus *S. punctatus* had RNA-guided dsDNA cleavage activity. Structurally, all three effectors adopt a bilobed architecture and recognize DNA duplexes as well as RNA–DNA heteroduplexes in a similar manner (Fig. 6). However, Cas12a possesses several domains that are missing in the corresponding regions of Fz and TnpB. The ωRNA scaffolds functionally substitute for some regions of Cas12a domains, such as the WED domain. This evolutionary relationship, characterized by the partial replacement of RNA with protein, parallels the transition observed in IscB and IsrB, in which ωRNA scaffolds are supplanted by protein components in their evolutionary descendant, Cas9 (refs. ^5,17,18^).

The biological role(s) of the RNA-guided endonuclease activity of Fzs remains unknown. In the case of TnpB, it seems to help TnpA-mediated transposition of the IS200/IS605 insertion sequences^4,6,15^. After TnpA-mediated peel-and-paste insertion, a TnpB-mediated double-strand break at the sister chromosome allows the insertion sequence to copy itself through homologous recombination. Some Fzs have been reported to co-occur with transposases with TnpA-like HUH endonuclease activity, such as Helitrons^7^, drawing a parallel with this peel-and-paste/cut-and-copy mechanism^15^. Thus, similar to TnpBs, it is possible that the RNA-guided DNA cleavage by Fzs acting at the sister chromosome could generally promote propagation of ssDNA transposons. Although we did not find any transposases flanked by the IRs of the *Spu-1* elements in the genome of *S. punctatus* (Supplementary Data 1 and 2), it is also possible that Fzs may help other transposons in *trans*. This is consistent with our observation of ghost loci in which the ωRNA is present but not the protein. In this scenario, a repertoire of guide sequences of ωRNAs, including those from ghost loci, could allow Fzs to target diverse sites, and their target transposons may not maintain their copy numbers over generations without Fz. Although transgene expression in *S. punctatus* has been reported^19,20^, more sophisticated genetic manipulation strategies to remove multiple copies of *Fz* genes in such eukaryotic host organisms would be required to answer this question. The loci of GtFz1, NlovFz2 and MmeFz2 seem to lack IRs, and no clear transposase association was detected in their genomes (Supplementary Data 3–5), indicating that Fzs may also be linked to a function distinct from that of TnpB. Further analysis of Fz IRs and gene associations may provide more insight into the biological function(s) of Fz.

**Extended Data Fig. 10:**
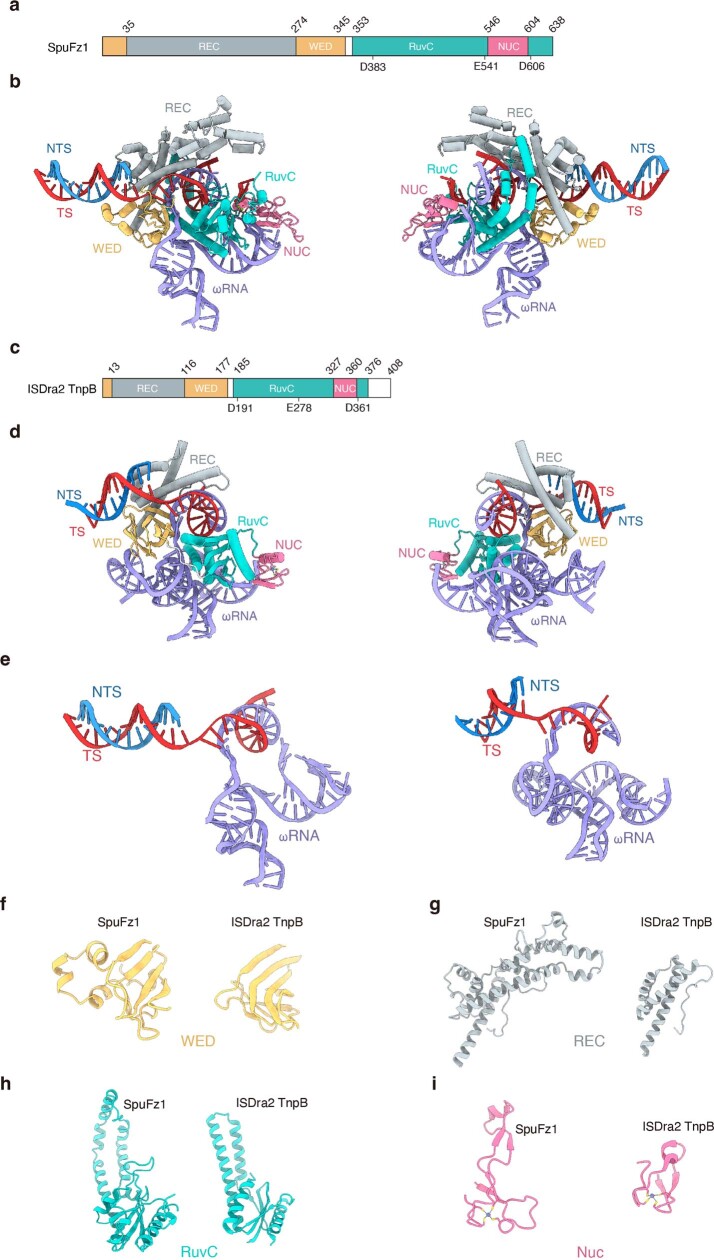
Structure comparison of SpuFz1 with ISDra2 TnpB. **a**, Domain architecture of SpuFz1. **b**, Overall structure of the SpuFz1–ωRNA–target DNA complex. **c**, Domain architecture of ISDra2 TnpB. **d**, Overall structure of the ISDra2 TnpB–ωRNA–target DNA complex (PDB code: 8H1J). Corresponding domains across structures are color-coded. **e**, Nucleic acid structure comparison. SpuFz1’s ωRNA lacks the pseudoknot structure inherent to ISDra2 TnpB. **f**, WED domain structure comparison. In contrast to the WED domain of ISDra2 TnpB, SpuFz1 exhibits three inserted small alpha helical structures, which provides interactions with TAM motifs of target DNA. **g**, REC domain structure comparison. An additional sequence of 136 aa is inserted within the REC domain of SpuFz1 relative to ISDra2 TnpB. **h**, RuvC domain structure comparison. The helices of SpuFz1’s RuvC domain are extended and interact with the additional part of the REC domain, providing enhanced structural protection for the RNA/DNA heteroduplex compared to ISDra2 TnpB. **i**, NUC domain structure comparison. Both SpuFz1 and ISDra2 TnpB share a conserved CCCC zinc finger motif in the NUC domain. The additional NUC structure in SpuFz1 aids in stabilizing the 5′ end of its ωRNA, which forms interactions with the RNA/DNA heteroduplex.

A comparison of the structures of SpuFz and ISDra2 TnpB shows the preservation of key mechanistic features during their evolution from prokaryotes to eukaryotes. For example, SpuFz1 and ISDra2 TnpB share a bilobed structure, consisting of the REC and NUC domains (Extended Data Fig. 10). However, there are differences between these proteins in the loading of ωRNA and target DNA. Specifically, the ωRNA of SpuFz1 lacks the pseudoknot structure, which is a common feature of TnpB and Cas12s (Extended Data Fig. 10). The three short alpha-helices present in the WED domain of the SpuFz1 structure are absent from the ISDra2 TnpB structure; this crucial segment facilitates the recognition of the DNA duplex by SpuFz1 (Extended Data Fig. 10f). The enlarged REC and RuvC domains of SpuFz1, coupled with the interactions between the ωRNA backbone and these domains (Extended Data Fig. 10g,h), provide enhanced protection for the RNA–DNA heteroduplex, in contrast to ISDra2 TnpB, in which a portion of the heteroduplex is exposed to solvent^8,11^. These structural insights deepen our understanding of the diverse mechanisms that these proteins use and provide valuable evolutionary perspectives. Future studies focusing on the structural differences between Fz1 and Fz2 will similarly advance our understanding of this protein family, and the evolution of their cognate ωRNA may reveal mechanistic differences between these variants.

From a bioengineering standpoint, the eukaryotic origin of Fz and its relatively small size compared with Cas9/12 make it an attractive starting point for further development. However, given the possible function of Fzs (and OMEGA effectors more broadly) in helping transposons propagate, they could be evolved for low activity and/or tightly regulated in their native organisms to prevent toxicity to the host. Reported engineering strategies for Cas12, such as systematic mutagenesis to introduce glycines^14,21^ and guide RNA engineering^22^, combined with in-depth screening of more Fzs could further improve their genome-editing performance. Nevertheless, we show here that the introduction of positively charged residues enhances activity, with our optimized SpuFz1-v2 achieving up to 18.4% indel activity on the human genome, highlighting the potential of Fzs as genome-editing tools.

## Methods

### Sequence mining of Fz protein

Multiple sequence alignments of Fzs were extracted from a previous article^7^, trimmed using trimAl v.1.2 (ref. ^23^) and converted into a hmm profile using HMMER v.3.3.2 (ref. ^24^). One sequence each of Fz1 and Fz2 was analysed with HHpred (web server), showing hits with HHpred probability greater than 90% for COG0675, PF07282 and PF01385. These three profiles and a custom profile made from Fz proteins were used as a seed for a hmmsearch to search for homologues in the NCBI non-redundant database (frozen in September 2022). Hits with a bit score equal to or greater than 20 were retained.

### Construction of a non-redundant eukaryotic structural database

The sequences of the 214 million models of eukaryotic protein contained in the AlphaFold EBI database^25^ were extracted and clustered at 50% of sequence identity and 50% of coverage with mmseqs2 v.12 (ref. ^26^). Taxids of each sequence extracted from this database were mapped to the NCBI taxonomy database to extract only structures from eukaryotic proteins. Finally, only predicted structures with at least 30 aa associated with a predicted local distance difference test (pLDDT) greater than 50 were selected to filter out low-quality predictions. The resulting database, which we named EukAFdb50, contains 11,693,265 predicted structures.

### Structural mining for Fz

Structural mining was performed using globular regions curated and extracted from predicted models (AlphaFold2 default parameters) of Fz1 and Fz2 and compared with EukAFdb50. The predicted structures were manually split into two main sets of seeds: the RuvC region and the WED–Rec region for each Fz. Each seed was compared with EukAFdb50 using Dali v.5 software with reciprocal comparison^27,28^, and hits with a score equal to or greater than 5 were curated using PyMOL v.1.2 (The PyMOL Molecular Graphics System, Schrödinger, LLC).

### Phylogeny of Fz

The 162,187 Fz homologous sequences (detected by sequence mining and curated from structural mining) were gathered and clustered at 50% of sequence identity and 50% of sequence coverages using mmseqs2 v.12 into 4,498 clusters. A representative of each group was extracted and aligned using Muscle v.5 software^29^ with the super5 algorithm. The resulting sequence alignment was trimmed with trimAl v.1.2 (gappyout)^23^ and curated manually using the Geneious platform. We mapped the catalytic site positions of the RuvC domain on the alignment and discarded sequences that harboured gaps in these positions, leading to a final alignment of 3,003 sequences. A structural model of each candidate was computed using AlphaFold2, and each structural model was compared with the structure of the RuvC domain of ISDra2 TnpB using Dali; 166 candidates did not align structurally to RuvC, indicating that they were potentially partial proteins (lacking the RuvC domain) or false positives detected during the profile mining (Extended Data Table 1). A tree was computed from the final alignment using IQtree^30^. The best model made on the final set was VT+T+R10, and bootstrap values were estimated with ultrafast bootstrap with 1,000 iterations.

### *Fz* loci analysis for Spu, Gt, Nlov and Mme

To identify all instances of Fz in Spu, all contigs from *S. punctatus DAOM BR117* were downloaded from NCBI. First, a translated blast^31^ was performed using a previously identified SpuFz^7^. Hits with e-value less than 0.05 were selected. IRs (right-end and left-end) seeds were extracted from the same Spu seed locus and used as inputs to search for ends in all contigs from Spu using blastn with a word length of 7. Hits were selected if their scores were greater than 20 and if they covered at least 17 nt. The distances between hits (Fz and end) in the genomes were calculated, and hits less distant than 25 kb were aggregated to form a locus. Loci that did not encompass IRs (at least two hits in inverted orientation) were manually curated to search for IRs using a motif search in the vicinity of a hit (up to 50 kb upstream and downstream) with the Geneious software. This analysis yielded 42 loci containing at least one Fz hit and 134 loci with at least one end. Assemblies downloaded from NCBI for *G. theta* CCMP2712, *N. lovaniensis* strain ATCC 30569 and *M. mercenaria* isolate YKG-2019 were analysed similarly.

### Cloning

Plasmids used in this study were cloned using general cloning methodologies including Gibson assembly with NEBuilder HiFi DNA Assembly Master Mix (New England Biolabs, E2621L) and Golden Gate assembly with a variety of type IIS restriction enzymes. The Stbl3 *Escherichia coli* strain (Thermo Fisher, C737303) was used for DNA cloning. The sequences of cloned constructs were confirmed by whole-plasmid sequencing following Tn5 tagmentation after mini-prep of plasmids^32^ with QIAprep reagents (QIAGEN, 27106). Human-codon-optimized Fz was cloned into two types of pCMV plasmid: one containing an N-terminal nuclear localization signal and HA tag, and the other containing a C-terminal HA and nuclear localization signal tag. Fz point mutants were constructed using site-directed mutagenesis with KLD Enzyme Mix (NEB, M0554S). The Fz ωRNA was optimized as follows. Secondary structures were predicted using mFold, and each scaffold region of the ωRNA variants was cloned under the U6 promoter with two inverted BbsI type IIS restriction sites behind the U6 promoter. Guides were cloned into the scaffolds by Golden Gate assembly as two annealed complementary oligonucleotides. For human genome targeting by Cas12a and Cas12f, vectors pY108 (no. 84739) and pCMV-AsCas12f1 (no. 171614) were obtained from Addgene.

### Fz RNP affinity purification

Fz orthologues were expressed in *S. cerevisiae* and affinity-purified. The Fz ORF and predicted 3′ IR regions were cloned under a GAL–GAPDH hybrid promoter in pRS426-URA3. A tag for protein purification (10xHis-MBP) was inserted between the start and second codons of Fz. The expression vector was transformed into a yeast BCY123 strain^33^ (a kind gift from the K. Nagai laboratory, MRC Laboratory of Molecular Biology, Cambridge) and selected on SD-URA plates. Colonies on half of the petri dish were scraped and transferred into a 50-ml starter culture of YM4 LMB media (0.67% yeast nitrogen base without aas, 0.5% casamino acids, 0.002% adenine and 0.002% tryptophan) supplemented with 2% raffinose for 17 h; this was used to inoculate 1 l of YM4 LMB media supplemented with 2% raffinose and 100 µg ml^−1^ ampicillin for growth at 30 °C with shaking at 180 rpm until an optical density at a wavelength of 600 nm of 1.0 was reached. Then, protein expression was induced in the presence of 2% galactose for 16 h. The cells were collected by centrifugation for 10 min at 4 °C at 4,000 rpm (Beckman Coulter Avanti J-E, rotor JLA8.100). The cell pellet was resuspended in 500 ml MQ water to remove residual media and pelleted again by centrifugation for 10 min at 4 °C at 4,000 rpm. All subsequent steps were performed at 4 °C. The cell pellet was resuspended in an equal volume of 2× lysis buffer (100 mM Tris-HCl, 500 mM NaCl, 2 mM MgCl_2_, 20 mM 2-mercaptoethanol, 2 mM imidazole and 10% glycerol, pH 8.0) supplemented with cOmplete ULTRA Tablets (Millipore Sigma 6538282001). The cell suspension was added dropwise into liquid nitrogen in an ice bucket, and the resulting frozen yeast beads were ground with a few pellets of dry ice in a prechilled coffee grinder (CG-618-SHARDOR). The frozen yeast powder was thawed and cleared by centrifugation for 15 min at 4 °C at 15,000 rpm (Beckman Coulter Avanti J-E, rotor JLA-16.25). The cleared lysate was applied to 1 ml of packed Ni-NTA (Qiagen) after its pH had been adjusted to 8.5 using Tris base and incubated with rotation for 1 h, followed by washing of the protein-bound resin in 100 ml lysis buffer. The resin was resuspended in 5 ml elution buffer (50 mM Tris-HCl, 250 mM NaCl, 1 mM MgCl_2_, 10 mM 2-mercaptoethanol, 500 mM imidazole and 5% glycerol, pH 8.0). The resulting elution was tested for the presence of the protein using NuPAGE (Invitrogen) and an eStain L1 Protein Staining System (GenScript). The protein was concentrated using an Amicon Ultra-15 centrifugal filter unit (50KDa NMWL, Millipore UFC905024) to 200 µl and used for downstream analysis. For the in vitro cleavage assays, Y-PER Yeast Protein Extraction Reagent (Thermo Fisher 78991) was used instead of the protein extraction process with the coffee grinder.

### Images of organisms

For micrographs and photographs, *S. punctatus* (Koch) Barr (ATCC 48900) and *N. lovaniensis* Steven et al. (ATCC 30569) were obtained from ATCC. *G. theta* Hill and Wetherbee was obtained from Bigelow (CCMP327). These organisms were cultured following the provider’s protocols. *M. mercenaria* was obtained from a fish market in Cambridge, MA.

### Small RNA-seq

*S. punctatus* (Koch) Barr (ATCC 48900) was grown following the provider’s protocol. The culture was spun down, and total RNA was extracted using a Direct-zol RNA kit (Zymo). Extracted RNA was treated with 10 units of DNase I (NEB) for 30 min at 37 °C to remove residual DNA and purified again with an RNA Clean & Concentrator-25 kit (Zymo). Ribosomal RNA was removed using a RiboMinus Transcriptome Isolation Kit, yeast (Thermo Fisher Scientific). The purified RiboMinus RNA was then treated with 20 units of T4 polynucleotide kinase (NEB) for 1 h at 37 °C, purified and treated with 20 units of 5′ RNA polyphosphatase (Lucigen) for 30 min at 37 °C and purified again. The purified RNA was used as input to an NEBNext Small RNA Library Prep for Illumina (NEB). Amplified libraries were gel-extracted and sequenced on an Illumina NextSeq with Read1 42 cycles, Read2 42 cycles and Index1 6 cycles. Adaptors were trimmed using CutAdapt v.2.4 and mapped to loci of interest using Bowtie2. For RNP RNA-seq, Fz RNPs were purified from *S. cerevisiae* as described. Concentrated RNP (100 µl) was used as input. The above protocol was followed without the RiboMinus Transcriptome Isolation Kit process.

### Fz RNP TAM screen

Purified Fz RNP and 25 ng of TAM library plasmid were supplemented with MgCl_2_, and the 10-µl reaction mixture (10 mM Tris-HCl, 50 mM NaCl, 5 mM MgCl_2_, 2 mM 2-mercaptoethanol and 1% glycerol, pH 8.0) was incubated at 37 °C for 4 h then quenched by addition of 10 µg RNase A (Qiagen) and eight units of proteinase K (NEB), each followed by a 15-min incubation at room temperature. DNA was extracted by PCR purification, and adaptors were ligated using an NEBNext Ultra II DNA Library Prep Kit for Illumina (NEB) using the NEBNext Adaptor for Illumina (NEB). Following adaptor ligation, cleaved products were amplified specifically using one primer specific to the TAM library backbone and one primer specific to the NEBNext adaptor by 12-cycle PCR using NEBNext High-Fidelity 2X PCR Master Mix (NEB) with an annealing temperature of 63 °C, followed by a second 18-cycle round of PCR to further add the Illumina i5 adaptor. Amplified libraries were gel-extracted and subjected to single-end sequencing on an Illumina MiSeq with Read1 80 cycles, Index1 8 cycles and Index2 8 cycles. TAMs were extracted, and an enrichment score for each TAM was calculated by filtering for all TAMs present more than once and normalizing to the TAM frequency in the input library. A position weight matrix based on the enrichment score was generated, and Weblogos (https://weblogo.berkeley.edu/logo.cgi) were visualized on the basis of this position weight matrix using a custom script.

### In vitro cleavage assays

dsDNA substrates were produced by PCR amplification of pUC19 plasmids or synthesized DNA fragments containing the target sites and the TAM sequences. Cy3- and Cy5-conjugated DNA oligonucleotides (IDT) were used as primers to generate the labelled dsDNA substrates. ssDNA substrates were ordered as Cy3-conjugated oligonucleotides (IDT). ssRNA substrates were transcribed in vitro using a HiScribe T7 Quick High Yield RNA synthesis kit (NEB) and purified using an RNA Clean and Concentrator-5 kit (Zymo). They were further labelled with pCp-Cy5 (Jena Bioscience) on their 3′ end. For the 3′ end labelling, 50 pmol ssRNA was incubated with 100 pmol pCp-Cy5 and 50 U T4 RNA ligase 1 (NEB) in reaction buffer at 16 °C for 24 h. Labelling reactions were purified using an RNA Clean and Concentrator-5 kit (Zymo). Through annealing of the ssRNA and its complement ssRNA, dsRNA substrates were prepared. Target cleavage assays were performed in a 10-µl reaction mixture containing 100 ng substrate and 2 µg protein in a final 1× reaction buffer of 25 mM Tris pH 8.0, 50 mM NaCl and 5 mM MgCl_2_. Assays were allowed to proceed at 37 °C for 2 h. Reactions were then treated with RNase A (Qiagen) and proteinase K (NEB) and purified using a PCR cleanup kit (Qiagen). For RNA substrates, RNase A treatment was skipped. For screening metal ions, MgCl_2_ was eliminated from the reaction buffer using an Amicon Ultra-0.5 Centrifugal Filter Unit, and the indicated metal was added. Collateral cleavage assays were performed using 100 ng of unlabelled dsDNA substrate along with 100 ng of Cy5.5-labelled collateral ssDNA, dsDNA, ssRNA or dsRNA substrates at 10 µl reaction volume. Purified DNA and RNA substrates after the assays were resolved by gel electrophoresis on E-gel 2% for dsDNA substrates or 15% TBE–urea polyacrylamide gels (Thermo Fisher Scientific) for ssDNA, dsRNA and ssRNA substrates.

### Mammalian cell culture and transfection

All transfection experiments were performed in the HEK293FT cell line (Thermo Fisher, R70007, authentication provided by the supplier; no mycoplasma testing was performed) grown in Dulbecco’s modified Eagle medium with high glucose, sodium pyruvate and GlutaMAX (Thermo Fisher, 35050061), further supplemented with 10% fetal bovine serum (VWR Seradigm, 89510-194). Transfections were performed with Lipofectamine 3000 (Thermo Fisher, L3000015) in 96-well plates unless otherwise noted. Cells were plated at approximately 2.0 × 10^4^ cells per well 16–20 h before transfection to ensure 90% confluency at the time of transfection. For each well on the plate, transfection plasmids (100 ng in total) were combined with OptiMEM Reduced Serum Medium (Thermo Fisher, 31985062) to a total volume of 5 µl and mixed with 0.2 µl P3000 reagent. Separately, 5 µl OptiMEM was combined with 0.3 µl Lipofectamine 3000 reagent. Plasmid and Lipofectamine solutions were then combined, incubated at room temperature for 10 min and pipetted on to cells.

### Human genome cleavage assay

For human genome cleavage assays, 2.0 × 10^4^ HEK293FT cells in 96-well plates were cotransfected with combinations of Fz expression plasmid (80 ng) and ωRNA expression plasmid (20 ng). After 3 days of incubation at 37 °C, the supernatant was removed and cells were resuspended in 40 µl QuickExtract DNA extraction solution (Lucigen, QE09050) and cycled at 65 °C for 15 min, 68 °C for 15 min, then 95 °C for 10 min to lyse cells. Two microlitres of lysate were used as the template for each 12.5 µl PCR reaction. Target sites were amplified with NEBNext High-Fidelity 2X PCR Master Mix (NEB, M0541L) under the following thermal cycling conditions: one cycle, 98 °C, 30 s; 15 cycles, 98 °C, 10 s, 65 °C, 20 s, 72 °C, 30 s; one cycle, 72 °C, 30 s; 4 °C hold. One microlitre of this first PCR product was used for the template for each 10-µl second PCR reaction: one cycle, 98 °C, 30 s; 15 cycles, 98 °C, 10 s, 63 °C, 20 s, 72 °C, 30 s; one cycle, 72 °C, 30 s; 4 °C hold (total 30 cycles for first and second PCR reactions). Amplicons were sequenced using a MiSeq Reagent Kit v.2, 300-cycle (Illumina, MS-102-2002). Indel efficiency was quantified using the established CRISPResso2 v.2.0.20b pipeline^34^.

### Preparation of the SpuFz1–ωRNA–target DNA ternary complex

The yeast cell pellet was resuspended in a buffer containing 20 mM HEPES pH 7.5, 150 mM NaCl, 2 mM MgCl_2_ and 4.5 mM TCEP supplemented with EDTA-free protease inhibitor cocktail (MedChem Express HY-K0010). The cell suspension was then added dropwise into liquid nitrogen in an ice bucket, and the resulting frozen beads were ground with a few pellets of dry ice in a prechilled coffee grinder (CG-618-SHARDOR). The frozen yeast powder was thawed and cleared by centrifugation for 35 min at 4 °C at 15,000 rpm (Beckman Coulter Avanti J-E, rotor JLA-16.25). The cleared lysate was applied to 3 ml of packed amylose resin (NEB), followed by washing of the protein-bound resin in 100 ml of buffer containing 20 mM HEPES pH 7.5, 150 mM NaCl, 2 mM MgCl_2_ and 4.5 mM TCEP. The MBP-tagged SpuFz1–ωRNA RNP was then eluted with the same buffer supplemented with 10 mM maltose (Sigma 6363-53-7) and concentrated using an Amicon Ultra-15 Centrifugal Filter Unit (50 kDa NMWL, Millipore UFC905024) to 400 µl. A 54-nt target DNA strand (TATTTGTAATTTGATTTCATAACCTATAGATATGCCCGGGTACCGAGCTCGAAT) and a 24-nt non-target DNA strand (ATTCGAGCTCGGTACCCGGGCATA) were purchased from GENEWIZ. For the reconstitution of the ternary complex, the purified RNP was mixed with the target DNA strand and non-target DNA strand at a molar ratio of 1:2:2 and incubated at 37 °C for 1 h. The sample was then loaded on a Superose 6 Increase 10/300 column (Cytiva) equilibrated with a buffer containing 20 mM HEPES pH 7.5, 150 mM NaCl, 2 mM MgCl_2_ and 4.5 mM TCEP. The eluted fractions of the ternary complex were pooled and concentrated for cryo-EM experiments.

### Cryo-EM grid preparation and data acquisition

Three microlitres of the purified SpuFz1–ωRNA–target DNA complex at around 3 mg ml^−1^ were applied to glow-discharged CryoMatrix R1.2/1.3 300-mesh gold holey grids with amorphous alloy film (Zhenjiang Lehua Technology Co., Ltd). The grids were blotted for 3 s at 100% humidity and 4 °C and then vitrified by plunging into liquid ethane using a Vitrobot Mark IV (Thermo Fisher Scientific). The prepared grids were then transferred to a EF-Krios (Thermo Fisher Scientific) operating at 300 kV with a GatanK3 imaging system collecting at ×105,000 nominal magnification. The calibrated pixel size of 0.4125 Å was used for processing. Videos were collected using Leginon 3.6 (ref. ^35^) at a dose rate of 28.56 e^−^/Å^2^/s with a total exposure of 1.80 s, for an accumulated dose of 51.41 e^−^/Å^2^. Intermediate frames were recorded every 0.03 s for a total of 60 frames per micrograph. A total of 8,727 images were collected at a nominal defocus range of 0.5–2.6 μm.

### Image processing and structure determination

Image processing was performed using CryoSPARC v.4.2.0 (ref. ^36^) and RELION 4.0 (ref. ^37^). Image stacks were subjected to beam-induced motion correction using MotionCor2.0 (ref. ^38^). Contrast transfer function parameters for each non-dose-weighted micrograph were determined using Gctf 1.18 (ref. ^39^). Automated particle selection yielded 3,455,057 particles, which were extracted on a binned dataset with a pixel size of 3.3 Å and subjected to reference-free two-dimensional classification, producing 945,611 particles with well-defined averages. These particles were re-extracted with a pixel size of 0.825 Å and subsequently subjected to Ab-initial reconstruction for three classes. The best subset showing clear structural features was subjected to heterogenous refinement for six rounds, producing a high-quality subset accounting for 501,142 particles. These particles were subsequently subjected to non-uniform refinement^40^, which generated a map with an indicated global resolution of 2.7 Å at a Fourier shell correlation of 0.143. DeepEMhancer^41^ was used to generate the sharpen map. The structure of the SpuFz1–ωRNA–target DNA ternary complex was determined using a model of SpuFz1 predicted by AlphaFold2 (refs. ^42,43^) and an ωRNA model predicted by RNAcomposer^44^ as initial models. The models were docked into the cryo-EM density maps using ChimeraX 1.4 (ref. ^45^), followed by iterative manual adjustment and rebuilding in ISOLDE 1.2 (ref. ^46^) and Coot 0.8.9 (ref. ^47^), against the cryo-EM electron density maps. Real space and reciprocal refinements were performed using PHENIX 1.18 (ref. ^48^). The model statistics were validated using MolProbity 4.5 (ref. ^49^). Structural figures were prepared in ChimeraX 1.4 and PyMOL (https://pymol.org/2/). The final refinement statistics are provided in Extended Data Table 1.

### Reporting summary

Further information on research design is available in the Nature Portfolio Reporting Summary linked to this article.

## Online content

Any methods, additional references, Nature Portfolio reporting summaries, source data, extended data, supplementary information, acknowledgements, peer review information; details of author contributions and competing interests; and statements of data and code availability are available at 10.1038/s41586-023-06356-2.

### Supplementary information

Supplementary Information Supplementary Fig. 1 and captions for Supplementary Tables 1–3 and Data 1–5.

Reporting Summary

Supplementary Table 1 Fz/TnpB.

Supplementary Table 2 List of reagents used in this study.

Supplementary Table 3 Fz orthologs assayed in this study.

Supplementary Data 1 Fz loci in *S. punctatus*.

Supplementary Data 2 Ghost Fz loci in *S. punctatus*.

Supplementary Data 3 Fz loci in *G. theta*.

Supplementary Data 4 Fz loci in *N. lovaniensis*.

Supplementary Data 5 Fz loci in *M. mercenaria*.

## Figures and Tables

**Extended Data Table 1:**
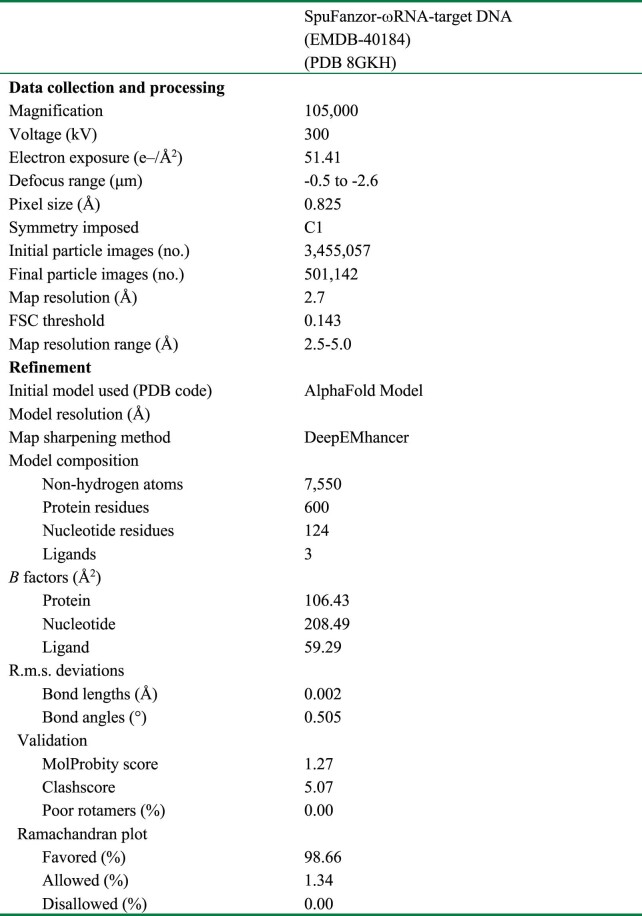
Cryo-EM data collection, refinement and validation statistics

## Data Availability

The phylogenetic tree is available on the iTOL website at the following address: https://itol.embl.de/tree/1378312326341663449344. The next-generation sequencing dataset containing small RNA-seq raw reads is available on SRA under BioProject PRJNA982412. The atomic coordinates of the SpuFz1–ωRNA–target DNA complex have been deposited in the Protein Data Bank with accession code 8GKH. The EM map of the SpuFz1–ωRNA–target DNA complex has been deposited in the Electron Microscopy Data Bank with accession code EMDB-40184.

## Code availability

A supporting Python script for analysis of TAM screens is publicly available at GitHub (https://github.com/MakotoSaito37/PAM-screen/blob/main/210304_PAMscreens_enrichment_analysis_updated.ipynb) and archived at Zenodo (10.5281/zenodo.8022520).
