# Supplementary material for: Fanzor is a eukaryotic programmable RNA-guided endonuclease
Source: Nature. 2023 Jun 28;620(7974):660–8. doi: 10.1038/s41586-023-06356-2 (PMC10432273; doi:10.1038/s41586-023-06356-2)
Supplement: Supplementary file 1 — Supplementary Fig. 1 and captions for Supplementary Tables 1–3 and Data 1–5. [file 41586_2023_6356_MOESM1_ESM.pdf]

nature portfolio

https://doi.org/10.1038/s41586-023-06356-2

Supplementary information

Fanzor is a eukaryotic programmable RNA-guided endonuclease

In the format provided by the authors and unedited

Supplementary Information for

Fanzor is a eukaryotic programmable RNA-guided endonuclease

Makoto Saito[1-5*], Peiyu Xu[1-5*], Guilhem Faure[1-5], Samantha Maguire[1-5], Soumya Kannan[1-5], Han Altae-Tran[1-5], Sam Vo[1-5], AnAn Desimone[1-5], Rhiannon K. Macrae[1-5], Feng Zhang[1-5†]

† Correspondence author: Feng Zhang, zhang@broadinstitute.org

This PDF file includes:

Supplementary Figure 1

Legend for Supplementary Table 1-3

Legend for Supplementary Data 1-5

a

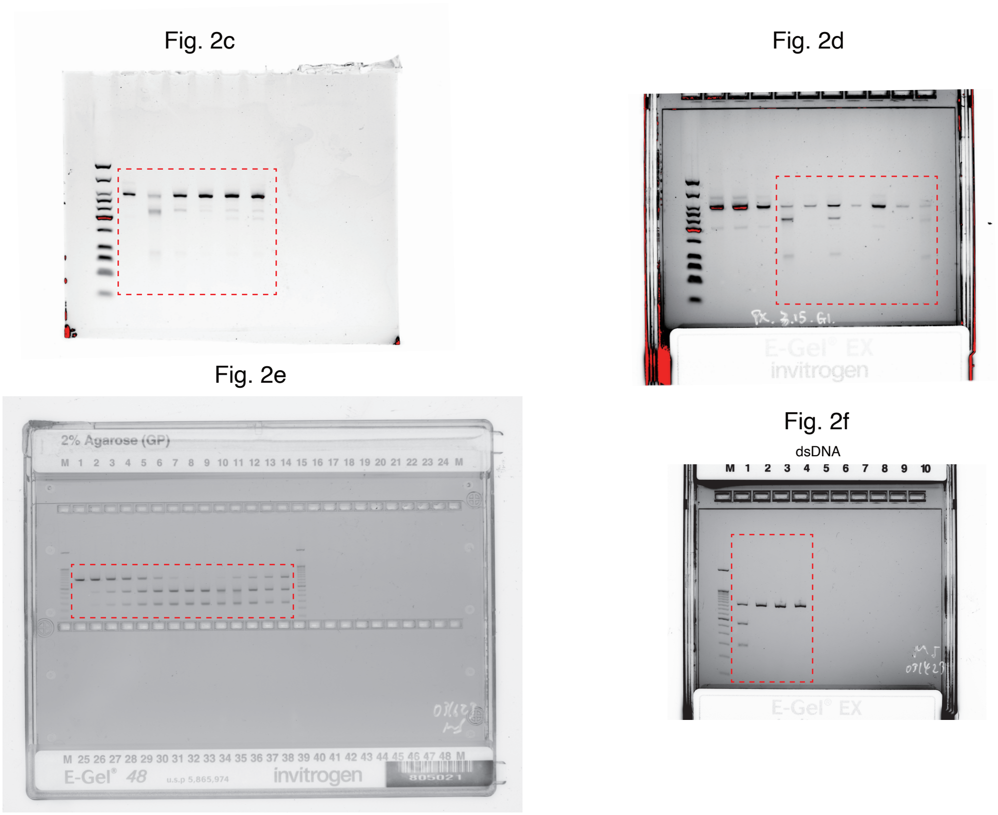

b

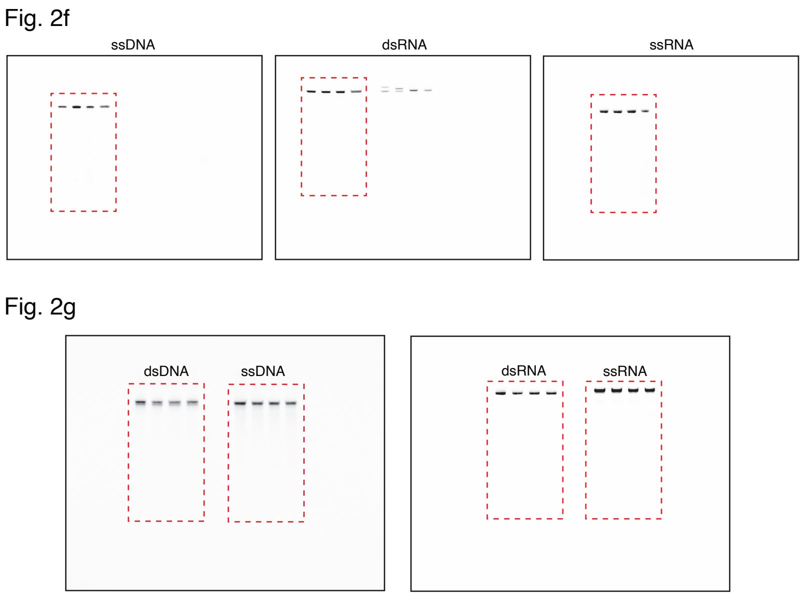

Supplementary Figure 1 I Uncropped gel images used in this study
a, Agarose gels (for Fig. 2c-f). b, TBE-Urea gels (for Fig. 2f and g).

Supplementary Table 1 | Fanzor/TnpB
Protein representatives from Fanzor/TnpB mining ordered from the phylogenetic tree. Fanzor1 and Fanzor2 branches are annotated in the first column and colored respectively in blue and pink. Eukaryotic radiations are colored in blue in the domain column. Additional information about protein length (in aa), Dali score extracted from the structural comparison with the RuvC domain of ISDra2 TnpB (greater than 3; N/A indicates no structural alignment detected by Dali), NCBI contig accession and coordinates on the contig (start, end, strand), NCBI protein accession number, domain, species and protein sequences are shown. For the viral Fanzors, putative host organisms are annotated based on literature searches.

Supplementary Table 2 | List of reagents used in this study.
Reagents include plasmids and primers.

Supplementary Table 3 | Fanzor orthologs assayed in this study.
Fanzor orthologs, types (Fz1 or 2), organism information, and related sequences (Fanzor ORF and predicted ωRNA) are listed.

Supplementary Data 1 | Fanzor loci in the *Spizellomyces punctatus*.
A genbank file contains DNA regions of the locus and annotation of IRs, Fz gene, ωRNA, and region of interest. CDS corresponds to automatic annotation from the NCBI database.

Supplementary Data 2 | Ghost Fanzor loci in the *Spizellomyces punctatus*.
A genbank file contains DNA regions of the locus and annotation of IRs, ωRNA, and region of interest.

Supplementary Data 3 | Fanzor loci in the *Guillardia theta*.
A genbank file contains DNA regions of the locus and annotation of IRs, Fz gene, ωRNA, and region of interest. CDS corresponds to automatic annotation from the NCBI database.

Supplementary Data 4 | Fanzor loci in the *Naegleria lovaniensis*.
A genbank file contains DNA regions of the locus and annotation of IRs, Fz gene, ωRNA, and region of interest. CDS corresponds to automatic annotation from the NCBI database.

Supplementary Data 5 | Fanzor loci in the *Mercenaria mercenaria*.
A genbank file contains DNA regions of the locus and annotation of IRs, Fz gene, ωRNA, and region of interest. CDS corresponds to automatic annotation from the NCBI database.
