# Supplementary material for: Fanzor is a eukaryotic programmable RNA-guided endonuclease
Source: Nature. 2023 Jun 28;620(7974):660–8. doi: 10.1038/s41586-023-06356-2 (PMC10432273; doi:10.1038/s41586-023-06356-2)
Supplement: Supplementary file 2 — Reporting Summary [file 41586_2023_6356_MOESM2_ESM.pdf]

nature portfolio

Corresponding author(s): Feng Zhang

Last updated by author(s): May 17, 2023

Reporting Summary

Nature Portfolio wishes to improve the reproducibility of the work that we publish. This form provides structure for consistency and transparency in reporting. For further information on Nature Portfolio policies, see our Editorial Policies and the Editorial Policy Checklist.

Statistics

For all statistical analyses, confirm that the following items are present in the figure legend, table legend, main text, or Methods section.

n/a	Confirmed	
☐	☒	The exact sample size (*n*) for each experimental group/condition, given as a discrete number and unit of measurement
☐	☒	A statement on whether measurements were taken from distinct samples or whether the same sample was measured repeatedly
☐	☒	The statistical test(s) used AND whether they are one- or two-sided<br>*Only common tests should be described solely by name; describe more complex techniques in the Methods section.*
☒	☐	A description of all covariates tested
☒	☐	A description of any assumptions or corrections, such as tests of normality and adjustment for multiple comparisons
☐	☒	A full description of the statistical parameters including central tendency (e.g. means) or other basic estimates (e.g. regression coefficient) AND variation (e.g. standard deviation) or associated estimates of uncertainty (e.g. confidence intervals)
☐	☒	For null hypothesis testing, the test statistic (e.g. *F*, *t*, *r*) with confidence intervals, effect sizes, degrees of freedom and *P* value noted<br>*Give P values as exact values whenever suitable.*
☒	☐	For Bayesian analysis, information on the choice of priors and Markov chain Monte Carlo settings
☒	☐	For hierarchical and complex designs, identification of the appropriate level for tests and full reporting of outcomes
☒	☐	Estimates of effect sizes (e.g. Cohen's *d*, Pearson's *r*), indicating how they were calculated

Our web collection on statistics for biologists contains articles on many of the points above.

Software and code

Policy information about availability of computer code

Data collection	Cyro-EM data were automatically collected using Leginon 3.6 on Titan Krios.
Data analysis	The following softwares were used in this study: CutAdapt v2.4, Bowtie 2, WebLogo (web server version), CRISPResso2 v2.0.20b, MotionCor2.1, Gctf v1.18, RELION 4.0-beta, UCSF ChimeraX 1.4, Phenix 1.18, Coot 0.8.9, Pymol 2.0.3.0, ISOLDE 1.2, CryoSPARC-v4.0, DeepEMhancer v0, MolProbity 4.5, AlphaFold 2.0., HHpred (web server version), MMseqs2 v12, Muscle v5, trimal 1.2, IQ-TREE v1.6.12, Geneious Prime 2019.1.3, Dali v5, BLAST v2.9.0, and HMMER 3.3.2.

For manuscripts utilizing custom algorithms or software that are central to the research but not yet described in published literature, software must be made available to editors and reviewers. We strongly encourage code deposition in a community repository (e.g. GitHub). See the Nature Portfolio guidelines for submitting code & software for further information.

Data

Policy information about availability of data

All manuscripts must include a data availability statement. This statement should provide the following information, where applicable:

- Accession codes, unique identifiers, or web links for publicly available datasets
- A description of any restrictions on data availability
- For clinical datasets or third party data, please ensure that the statement adheres to our policy

Phylogenetic tree is available on itol website at the following link address: https://itol.embl.de/tree/1378312326341663449344. Next-Generation Sequencing data

set containing small RNA-seq raw reads is available on SRA under BioProject PRJNA982412. The atomic coordinates of the SpuFz1-ωRNA-target DNA complex has been deposited in the Protein Data Bank (PDB) with the accession code 8GKH. The EM map of SpuFz1-ωRNA-target DNA complex has been deposited in the Electron Microscopy Data Bank (EMDB) with accession code EMDB-40184.

Human research participants

Policy information about studies involving human research participants and Sex and Gender in Research.

Reporting on sex and gender	N/A
Population characteristics	N/A
Recruitment	N/A
Ethics oversight	N/A

Note that full information on the approval of the study protocol must also be provided in the manuscript.

Field-specific reporting

Please select the one below that is the best fit for your research. If you are not sure, read the appropriate sections before making your selection.

☒ Life sciences ☐ Behavioural & social sciences ☐ Ecological, evolutionary & environmental sciences

For a reference copy of the document with all sections, see nature.com/documents/nr-reporting-summary-flat.pdf

Life sciences study design

All studies must disclose on these points even when the disclosure is negative.

Sample size	Experiments were done with independent biological samples (usually n≥3) and highly comparable results were obtained. Our sample sizes (usually n≥3) were chosen according the standards of our lab (e.g. Saito et al., Cell, 2021 and Faure et al., Mol. Cell, 2023) and similar to the standard of other labs in the field.
Data exclusions	No data was excluded.
Replication	All attempts at replication were successful in ≥3 experiments with independent biological samples. The methods and reagents used are described in detail.
Randomization	Each sample is allocated to each experimental condition by experimenters, and thus no randomization was used.
Blinding	All attempts at replication were successful in ≥3 experiments with independent biological samples, and the significance was statistically confirmed. Thus, blinding was not applied.

Reporting for specific materials, systems and methods

We require information from authors about some types of materials, experimental systems and methods used in many studies. Here, indicate whether each material, system or method listed is relevant to your study. If you are not sure if a list item applies to your research, read the appropriate section before selecting a response.

Materials & experimental systems

n/a	Involved in the study	
☒	☐	Antibodies
☐	☒	Eukaryotic cell lines
☒	☐	Palaeontology and archaeology
☒	☐	Animals and other organisms
☒	☐	Clinical data
☒	☐	Dual use research of concern

Methods

n/a	Involved in the study	
☒	☐	ChIP-seq
☒	☐	Flow cytometry
☒	☐	MRI-based neuroimaging

Eukaryotic cell lines

Policy information about cell lines and Sex and Gender in Research

Cell line source(s)	293FT Cell Line from ThermoFisher Scientific.
Authentication	Cells (obtained new for this study) were authenticated by the supplier.
Mycoplasma contamination	No mycoplasma contamination was confirmed by the authors.
Commonly misidentified lines (See ICLAC register)	No commonly misidentified cell lines were used.
